# Nutrition and Exercise Knowledge, Attitude, and Practice: A Scoping Review of Assessment Questionnaires in Cancer Survivorship †

**DOI:** 10.3390/nu17091412

**Published:** 2025-04-23

**Authors:** Zhi Qi Hoe, Ria Joseph, Narayanee Dick, Christina Syu Hong Thio, Matthew Wallen, Ling Di Chua, Claire Miller, Jane Lee, Raymond Javan Chan, Chad Yixian Han

**Affiliations:** 1 Department of Dietetics, National University Hospital, National University Health System, Singapore 119074, Singapore; zhi_qi_hoe@nuhs.edu.sg; 2 Caring Futures Institute, College of Nursing and Health Sciences, Flinders University, Adelaide, SA 5042, Australia; ria.joseph@flinders.edu.au (R.J.); yani.dick@flinders.edu.au (N.D.).matthew.wallen@flinders.edu.au (M.W.); jane.lee.714@gmail.com (J.L.); raymond.chan@flinders.edu.au (R.J.C.); 3 Department of Nutrition and Dietetics, Tan Tock Seng Hospital, National Healthcare Group, Singapore 308433, Singapore; silver_ld_chua@ttsh.com.sg; 4 Healthy Lifestyles Australia, Lowood, QLD 4311, Australia; clairem@hla.au

**Keywords:** nutrition, exercise, knowledge, attitude, practice, assessment, cancer survivors

## Abstract

Cancer survivors are encouraged to maintain a healthy diet and engage in regular exercise to improve overall physical and psychosocial health, and to reduce the likelihood of cancer recurrence and related mortality. Consequently, nutrition and exercise (the structured component of physical activity) knowledge, attitude, and practice (NE-KAP) are frequently evaluated in research studies involving cancer survivors and are associated with various health outcomes. The aims of this scoping review were to (1) systematically map the types of NE-KAP assessment questionnaires used in cancer survivorship research (i.e., trend or use over the past five years), (2) provide a directory of questionnaires, and (3) identify the most frequently reported health outcomes that have been associated with them. A systematic search was conducted across four databases—Medline, Emcare, CINAHL, and Scopus—from 1 January 2019 to 9 May 2024, for studies addressing one or more aspects of NE-KAP (e.g., food frequency questionnaire for nutrition practice, attitude towards exercise scale for exercise attitude). Eligible studies were extracted, assessed, and reviewed by two independent authors, and data were summarized descriptively. Of the initial 5452 records screened, 1122 articles were screened for full text, and 852 were deemed eligible, with 262 studies included. There was an overall increasing trend in NE-KAP research in cancer survivorship research from 2019 to 2023. Of the 200 unique questionnaires aligning with at least one NE-KAP domain, 45 were untitled and created/adapted specifically for their respective studies, with limited information about their psychometric properties. Out of the 262 included studies, the most utilized questionnaires were those measuring nutrition or physical activity practices, such as study-specific food frequency questionnaires (*n* = 26, 10%) and the Godin–Shephard Leisure–Time Physical Activity questionnaire (*n* = 52, 20%). Out of studies that had reported health outcomes (nutrition, *n* = 23; exercise, *n* = 40), health-related quality of life was most commonly associated with nutrition (*n* = 12, 53%) and exercise (*n* = 9, 23%), and from cross-sectional studies (nutrition, *n* = 13; exercise, *n* = 23). An emphasis was placed on assessing nutrition and exercise practices, with limited attention towards the knowledge and attitude domains. Psychometric evaluation of questionnaires was also lacking.

## 1. Introduction

Cancer care has advanced significantly, leading to improved survivor rates and life expectancy for cancer survivors globally [1,2,3]. Alongside these advancements, the perception of cancer has transitioned from being predominantly associated with mortality to optimism about long-term survivorship [1,2,3,4]. In 2022, there were over 53 million people living with cancer 5 years beyond diagnosis globally, which was more than double compared to 2002 [5,6].

Cancer survivorship is typically defined as the period beginning at the time of cancer diagnosis and continuing throughout the individual’s lifespan [7]. Regardless, surviving cancer presents new challenges. For example, during cancer treatments, nutrition may be compromised due to increased physiological requirements from stress or reduced appetite caused by treatment, often leading to accelerated weight loss [8]. Following active treatment, cancer survivors are at increased risk of developing secondary malignancies, long-term treatment side effects, metabolic and cardiovascular complications, obesity, and mental health issues, all of which require a change in lifestyle and ongoing supportive care [2,3,4,9]. Despite treatment advancements, survivors continue to experience physical limitations such as fatigue, reduced mobility, sarcopenia, and increased body fat, all of which negatively affect their health and psychosocial outcomes [10,11].

Many supportive care issues faced by cancer survivors are related to—or impacted by—diet and physical activity, which motivates them to seek information and address concerns related to food choices and exercises [12]. Much of this knowledge-seeking behavior stems from the range of advice (often conflicting) that cancer survivors receive to what seemed like simple questions for people without a history of cancer, e.g., what foods to eat, what supplements to take, or if they should exercise [13]. For instance, cancer survivors are often advised to either rest or engage in exercise to combat fatigue. This can be confusing because resting alone may not effectively alleviate cancer-related fatigue, and the condition itself can pose a significant barrier to physical activity [14]. Cancer survivors have also reported dietary issues such as food avoidance and attempts at alternative unproven dietary strategies, e.g., restrictive diets and herbal remedies [15].

The adoption of regular physical activity is observed to reinforce positive outcomes to alleviate the individual’s negative mood and reduce stress [16]. Embracing healthy eating habits and engaging in regular physical activity can therefore play a key role in reducing the risk of developing comorbidities in cancer survivors [16]. This focus on nutrition and physical activity has been reflected in several practice guidelines, e.g., the World Cancer Research Fund (WCRF) recommends 150 min of moderate-intensity exercise or 75 min of vigorous exercise per week and prioritizing plant-based foods; the National Comprehensive Cancer Network (NCCN) and the American Society for Clinical Oncology (ASCO) highlight the importance of maintaining a healthy dietary pattern and being as physically active as possible [17,18].

**Figure 1:**
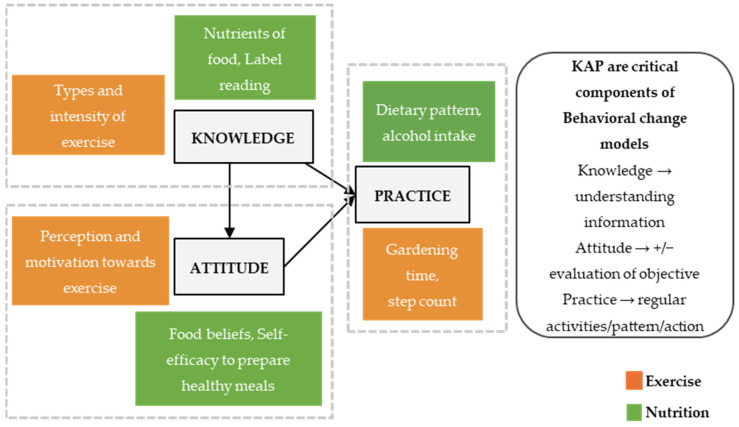
Conceptual diagram and relationship of nutrition–exercise knowledge–attitude–practice (NE-KAP) with examples, adapted from convention KAP model [31].

To effectuate nutrition and physical activity interventions that benefit cancer survivors, researchers often focus on nutrition and physical activity practices as outcomes and compare them with health indicators either cross-sectionally or over a time period, such as fruit intake versus body mass index and sitting time versus fatigue severity [19]. However, it is imperative to go beyond the measurement of practice alone and also examine the knowledge and attitudes that influence these practices or behaviors [20]. The Knowledge–Attitude–Practice (KAP) model is a widely used conceptual framework in healthcare and behavioral modification programs [20]. People equipped with the required knowledge can positively impact their attitudes and consequently influence the alteration of their practices or behaviors (Figure 1) [21,22]. This shift in mentality, coupled with newly acquired knowledge, can be leveraged to drive sustainable lifestyle changes [23,24,25]. Understandably, various assessments measuring the effectiveness of interventions are focused on practice (outcome), as evaluating knowledge and attitudes accurately remains challenging as the range of questionnaires available can be overwhelming or those commonly used may not be validated for specific study populations [26]. In addition, researchers face several challenges when selecting assessment questionnaires to evaluate such changes in nutrition or exercise knowledge, attitude, and practice after an intervention. They are required to assess the validity, reliability, and sensitivity of the questionnaires, ensuring they are appropriate for the target population and context, and review existing validation studies to confirm their suitability [27,28]. Nutrition and physical activity recommendations during and after cancer treatment can also vary across regions and cultural contexts. Hence, questionnaires developed in one setting may not account for local dietary and physical activity patterns, cultural beliefs about cancer, or available resources [29,30].

The aim of this scoping review was to (1) systematically map the types of Nutrition Exercise–Knowledge Attitude Practice (NE-KAP) assessment questionnaires used in cancer survivorship research (i.e., trend or use over the past five years), (2) provide a directory of questionnaires, and (3) identify the most frequently reported health outcomes that have been associated with them.

## 2. Materials and Methods

### 2.1. Protocol and Registration

This study used a systematic approach for scoping reviews and was reported in accordance with the Preferred Reporting Items for Systematic Reviews and Meta-Analyses Extension for Scoping Reviews (PRISMA-ScR) checklist (Supplementary Materials, Table S1) [32].

### 2.2. Aims and Methodology

The aims and methods were prospectively documented in the Open Science Framework Registry—https://osf.io/k2fm4, accessed on 27 October 2023. The search strategy (Supplementary Materials, Table S2), consisting of keywords and controlled vocabulary terms, was developed by an author (C.Y.H.) and an academic librarian and was based on search strategies agreed upon by the lead authors. Reference lists of eligible full-text English articles were also screened. An academic librarian conducted systematic searches of peer-reviewed literature across four electronic databases (Medline, Emcare, CINAHL, Scopus) from 1 January 2014 up to 9 May 2024. Given the aim of the study was to provide a contemporary report on the trend of use and the large number of studies found, the scope period was limited to the past 5 years between 1 January 2019 to 31 December 2023. The sources yielded from the search were imported into Covidence software (www.covidence.org, accessed on 9 May 2024). Screening and selection of articles were conducted independently by five authors (C.Y.H., Z.Q.H., L.D.C., R.J., and N.D.) via Covidence software using the study inclusion and exclusion criteria (Table 1) [33]. Discrepancies regarding the inclusion of articles were resolved through discussion.

Data extraction was performed using a predefined data extraction form, which included: “Questionnaires measuring Nutrition KAP”, “Questionnaires measuring Exercise KAP”, “Number of questions (in questionnaires)”, “Health Outcome associated with the results from questionnaires (if any)”, “Any referrals after assessment”. These were collectively revised from a previous scoping review by four authors (C.Y.H., Z.Q.H., L.D.C., and N.D.) [35]. Extracted data was reviewed by two authors (C.Y.H and Z.Q.H.). In line with the aim of this scoping review, the intention of the review was to map and summarize available evidence. Findings of interest to this scoping review (i.e., types of assessment questionnaire, population group used, associated health outcome measures) were narratively summarized.

For this review, a unique questionnaire was defined as a systematically designed or adapted instrument that evaluates individuals’ understanding, perceptions, and behaviors related to nutrition and/or exercise. This questionnaire would have integrated objective metrics (i.e., number/grade) to measure: (1) Knowledge—the extent of factual and conceptual understanding of nutrition and exercise principles; (2) Attitude—beliefs, perceptions, and motivation towards nutrition and exercise practices; (3) Practice—the actual implementation of nutrition and exercise behaviors in daily life.

## 3. Results

Following the removal of duplicates, 5452 records remained, of which 4330 were excluded after title and abstract screening. Of the 1122 full-text articles assessed for eligibility, 852 were eligible, and a total of 262 were between the period of interest and included in the narrative synthesis (Figure 1). Due to the extensive list, the references of the included studies were presented in Supplementary Materials, File S1.

### 3.1. Characteristics of Included Studies (Cancer and Study Types) and 5-Year Publication Trend

**Figure 2:**
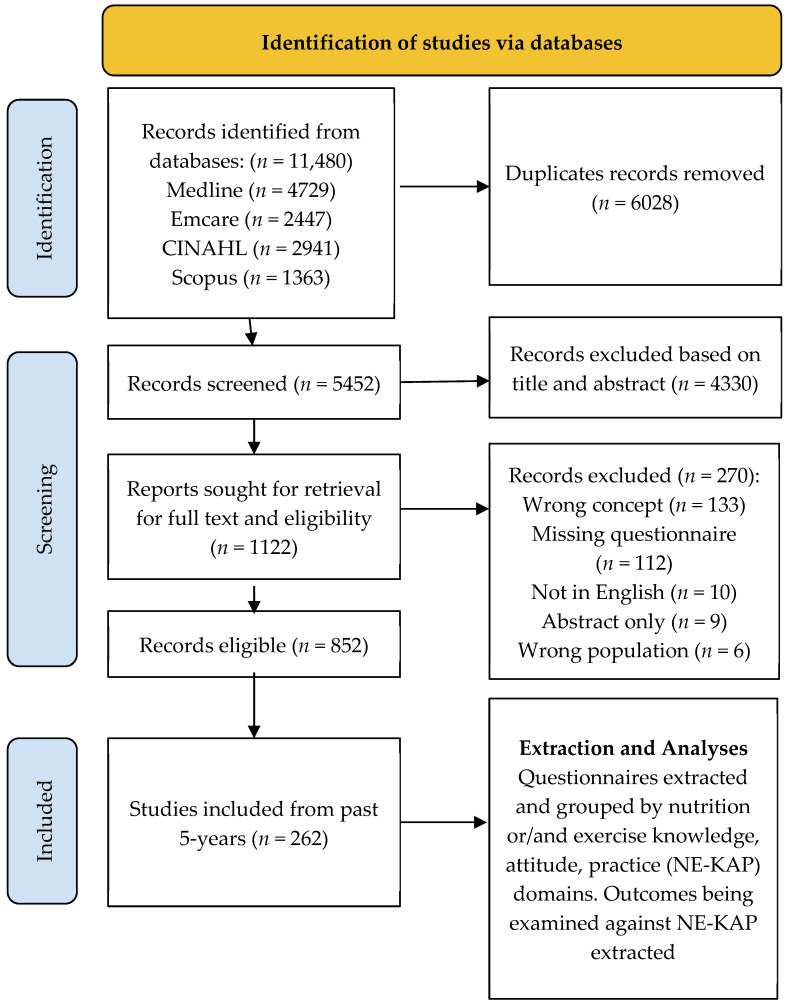
PRISMA 2020 flow chart illustrating results of the search and study selection process.

There was a substantial number of peer-reviewed publications on NE-KAP within the cancer survivorship research domain published annually. These publications have shown an increasing trend over the 5-year period (i.e., 2019 to 2023) and doubling from 2022 to 2023 (Figure 2).

**Figure 3:**
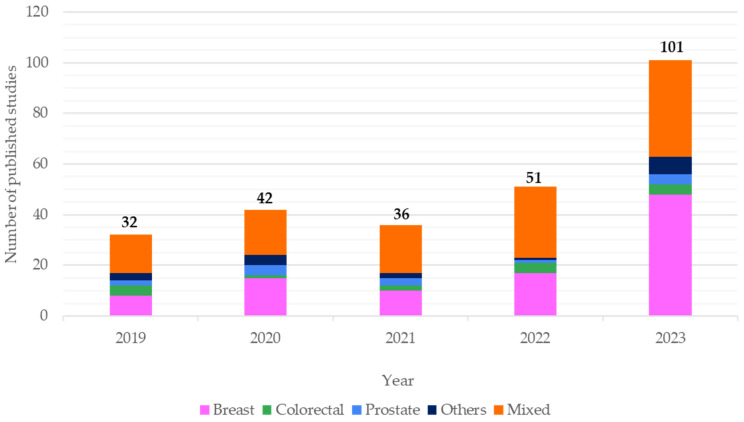
Trend of the number of studies (*n* = 262) and types of cancer cohort in these studies published between 2019 to 2023 in cancer survivorship that measured nutrition–exercise knowledge, attitude, and/or practice; others—endometrial; head and neck; hematological; lymphoma; myeloma; ovarian; pancreatic; stomach.

The majority of the study cohort included a mix of different cancer types or breast cancer only (Figure 3). One hundred and fifty-seven studies included more than one cancer type within their cohort (e.g., endometrial, cervix, bladder, lung, kidney). Most of these were cross-sectional studies (*n* = 88), followed by RCTs (*n* = 24), prospective cohort studies (*n* = 13), mixed-methods evaluation (*n* = 7), and single-arm intervention studies (*n* = 3). For studies involving singular cancers, breast cancer survivors were most commonly reported in randomized controlled trials (RCT) (*n* = 29), observational cohort studies (*n* = 18), cross-sectional surveys (*n* = 37), single-arm intervention studies (*n* = 4) and mixed-methods evaluation (*n* = 3).

The list of NE-KAP questionnaires found from the included studies was tabulated (Table 2). Only one (2015 French Cancer Barometer Survey) out of the 155 titled and 45 untitled assessment questionnaires included questions for all three domains, i.e., knowledge, attitude, and practice in both Nutrition and Exercise [36,37]. Thirteen assessment questionnaires measured at least two out of three domains: (1) 2015 French Cancer Barometer Survey; (2) Health Education Impact Questionnaire; (3) Exercise Processes of Change Questionnaire; (4) Health Action Process Approach Constructs Questionnaire; (5) Three-Factor Eating Questionnaire; (6) Preferences and Self-Efficacy of Diet and Physical Activity Behaviors Questionnaire for Latina Women; (7) Acceptance and Action Questionnaire-II; (8) Basic Psychological Needs in Exercise Scale; (9) Behavioral Regulation in Exercise Questionnaire; (10) Theory of Planned Behavior Questionnaire; and (11–13) three untitled study-specific questionnaires [38,39,40,41]. The nutrition and exercise practice domain was more commonly researched compared to knowledge and attitude (Table 2).

*Knowledge*—We identified three nutrition–knowledge assessment questionnaires, while one study used a study-specific online questionnaire to collect data on nutrition knowledge, food preferences, and intake frequency [41]. There were seven assessment questionnaires and four untitled, study-specific questionnaires that measured exercise–knowledge (Table 2).

*Attitude*—We identified 18 assessment questionnaires and five untitled, study-specific questionnaires that measured nutrition–attitude, and 39 assessment questionnaires and 15 study-specific questionnaires that measured exercise–attitude (Table 2).

*Practice*—We identified 47 assessment questionnaires and 14 untitled, study-specific questionnaires that measured nutrition–practice, and 55 assessment questionnaires and 22 untitled study-specific questionnaires that measured exercise–practice (Table 2).

Across the three domains, about a quarter of the unique questionnaires were untitled, study-specific questionnaires (*n* = 55, 27%) created by authors to meet the specific objectives of their study, with limited or no information on their psychometric evaluation/properties. These questionnaires varied in size (i.e., ranging from a single question to as many as 48, with some containing multiple parts to single questions). They were either developed by the authors or adapted from one or more existing validated questionnaires to address the unique research question and population in their study. The most common example of such adapted questionnaires is the Food Frequency Questionnaire (FFQ), used to measure nutrition–practice. For example, we identified a study that used a questionnaire amalgamated from three different validated and non-validated questionnaires [39].

### 3.2. Nutrition Knowledge, Attitude, and/or Practice Assessment Questionnaires

**Figure 4:**
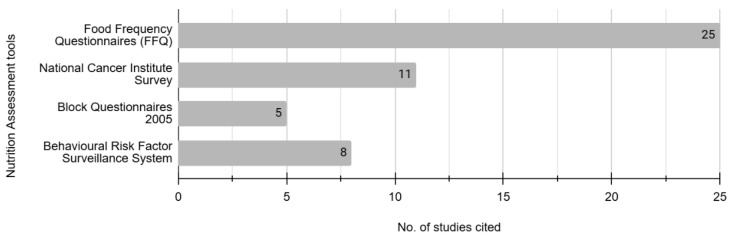
Top four nutrition–KAP assessment questionnaires used in cancer survivorship research.

The top four assessment questionnaires used widely in studies were used to measure nutrition–KAP (Figure 4). Most studies (*n* = 26) developed and used an FFQ, which measured only nutrition–practice. Eleven studies used the National Cancer Institute (NCI)’s survey, five studies used Block Questionnaires, and eight studies derived Nutrition–KAP from the Behavioral Risk Factor Surveillance System (BRFSS).

#### 3.2.1. Food Frequency Questionnaires (*n* = 25) [64,68,69,83,84,98,99,100,101,102,103,104,105,106,107,108,109,110,111,112,113,114,115,116,117]

These FFQs, which may or may not be validated, consisted of a median of 110 items, and ranged from 12 to 204 question items. These questionnaires can be self- or interviewer-administered and measure the frequency at which participants consume specific food or beverage items (e.g., “How often do you eat apples, pears, or bananas?” with option answers such as “Never or < 1 time/month”, “1–3 times/month”, “1 times/week”, “2–4 times/week”, “1 time/day”, “2–3 times/day”, “4+ times/day”). These questionnaires were then used to evaluate dietary patterns and/or macro- and micronutrient intake.

#### 3.2.2. National Cancer Institute Surveys (*n* = 11) [61,76,118,119,120,121,122,123,124,125,126]

Several surveys and questionnaires from the NCI survey were used to assess nutrition–KAPs. The Food Attitudes and Behaviors Survey comprises eight sections and a total of 65 questions exploring attitudes and beliefs about food, general health, shopping habits, fruit and vegetable consumption, eating behaviors, and food preferences. Another nutrition–practice assessment questionnaire identified in our review was the Diet History Questionnaire III, a web-based questionnaire designed to evaluate an individual’s dietary practices using a list of 135 food and beverage items along with 26 dietary supplement questions. The Health Information National Trends Survey (HINTS) consists of two questions specifically aimed at identifying fruit and vegetable consumption.

#### 3.2.3. Block Questionnaires (*n* = 5) [127,128,129,130,131]

The questionnaire consists of 110 food items and is designed to be self- or interviewer-administered. The validity and reliability of the questionnaire have been assessed in multiple studies [132,133]. Similarly to the FFQ, it captures both frequency and portion size of food intake. It is also available in Spanish and can be administered in either paper or electronic forms.

#### 3.2.4. Behavioral Risk Factor Surveillance Survey (*n* = 8) [134,135,136,137,138,139,140,141]

The Behavioral Risk Factor Surveillance Survey is a validated national-level telephone survey comprising 10–12 items identifying fruit and vegetable intake. An example question: “*During the past month, not counting juice, how many times per day, week, or month did you eat fruit? Count fresh, frozen, or canned fruit*”. From our findings, three studies used this survey in breast cancer survivors, while one study used it in prostate cancer survivors [136,138,139,142].

### 3.3. Exercise Knowledge, Attitude, and/or Practice Assessment Questionnaires

**Figure 5:**
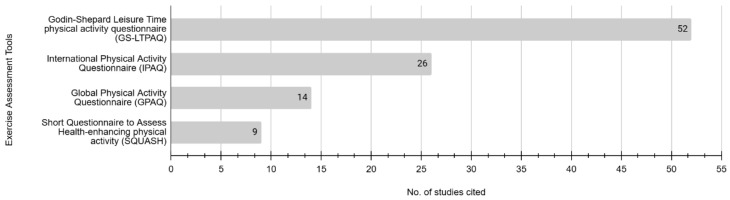
Top four Exercise–KAP assessment questionnaires used in cancer survivorship research.

The top four assessment questionnaires widely used in studies to measure exercise–practice, as shown in Figure 5, were the Godin–Shephard Leisure–Time Physical Activity Questionnaire, the International Physical Activity Questionnaire (IPAQ), the Global Physical Activity Questionnaire (GPAQ), and the Short Questionnaire to Assess Health-Enhancing Physical Activity (SQUASH). These four questionnaires measured only one of the three domains (Practice).

#### 3.3.1. Godin-Shephard Leisure–Time Physical Activity Questionnaire (*n* = 52) [39,44,46,47,49,54,60,61,62,63,64,65,67,83,120,124,125,126,143,144,145,146,147,148,149,150,151,152,153,154,155,156,157,158,159,160,161,162,163,164,165,166,167,168,169,170,171,172,173,174,175,176,177]

Most studies (*n* = 52) used the Godin–Shephard Leisure–Time Physical Activity Questionnaire (GS-LTPAQ). It is a quick, self-report, validated questionnaire used to assess an individual’s free-time physical activity levels. It consists of four questions: three measuring the frequency of strenuous, moderate, and light exercise per week, and one assessing whether the individual regularly engages in activity intense enough to induce sweating. Following that, a Leisure Score Index is calculated by weighting each activity type, classifying individuals as active, moderately active, or insufficiently active based on their total score. Studies that used the GS-LTPAQ included cohorts that had a mix of different cancers, but singular cancer cohorts were most frequently reported—breast cancer survivors only [39,47,143,144,152,156,161,165,166,178,179], colorectal cancer survivors only [83,145], followed by prostate cancer survivors only [65,128,162].

#### 3.3.2. International Physical Activity Questionnaire (*n* = 26) [40,70,75,87,180,181,182,183,184,185,186,187,188,189,190,191,192,193,194,195,196,197,198,199,200,201]

Developed to provide a standardized way for assessing physical activity levels across different populations and countries, the International Physical Activity Questionnaire (IPAQ) measures physical activity in adults (not specific to cancer survivors). There are two versions—short (7 questions) and long (27 questions)—which assess different types of physical activity—vigorous, moderate, walking, and sitting—over the past seven days. Based on the responses, individuals are categorized into low, moderate, or high levels of physical activity. The majority of the studies used the shorter version of the questionnaire.

#### 3.3.3. Global Physical Activity Questionnaire (*n* = 14) [54,58,110,202,203,204,205,206,207,208,209,210,211]

Developed by the World Health Organization (WHO), the Global Physical Activity Questionnaire (GPAQ) is designed to measure physical activity levels across different populations. It consists of 16 questions that assess physical activity in three areas—work, travel (walking/cycling), and leisure time. There is also an additional question that measures sedentary behavior, based on how much time a person spends sitting each day. In our review, we identified a Korean version, which was reported three times [58,210,211].

#### 3.3.4. Short Questionnaire to Assess Health-Enhancing Physical Activity (*n* = 9) [69,117,212,213,214,215,216,217]

The Short Questionnaire to Assess Health-Enhancing Physical Activity (SQUASH) was designed to measure physical activity levels by examining four key domains: commuting, work, household tasks, and leisure–time activities [37]. Instead of a fixed number of questions like the above-mentioned questionnaires, it uses a flexible format where respondents report the type, frequency, and duration of activities they typically engage in a week, which is then used to estimate a person’s total physical activity level.

### 3.4. Health Outcomes Associated with Nutrition–Exercise Knowledge, Attitude, and/or Practice Assessment Questionnaires

Out of the 262 included studies, 23 and 40 studies reported health outcomes associated with nutrition and exercise, respectively. Health-related quality of life was most commonly associated with nutrition (*n* = 12, 53%) and exercise (*n* = 9, 23%). Studies comparing nutrition–KAP with a health outcome were most commonly cross-sectional (*n* = 13), followed by interventional (*n* = 5; randomized controlled trials (*n* = 3) or single-arm interventional (*n* = 2)) and two prospective cohort studies. Studies comparing exercise–KAP with a health outcome were most commonly cross-sectional (*n* = 23), followed by interventional (*n* = 7; randomized controlled trials (*n* = 7) or single-arm interventional (*n* = 1)) and prospective cohort studies (*n* = 6).

## 4. Discussion

To the authors’ knowledge, this is the first review of NE-KAP assessment questionnaires used in cancer survivorship research [218]. We identified a significant diversity in the assessment questionnaires used across studies, with a total of 200 unique questionnaires being reported. The wide variability and lack of standardization around the use of these questionnaires in cancer survivors makes it difficult to compare findings across different studies, limiting the ability to draw consistent conclusions. Notably, over 27% of the questionnaires were untitled, tailored to specific studies, and lacked psychometric evaluation. This raises significant concerns about the reliability and generalizability of their findings. Additionally, the introduction of these new questionnaires may exemplify the duplication of efforts frequently observed in research. For novice researchers, it can be particularly confusing when different questionnaires have similar names or when the same questionnaire is referred to by different names, such as GS-LTPAQ. This issue is compounded if they are not familiar with the domain, leading to potential misunderstandings and errors. The review also revealed that most studies emphasized assessing nutrition and exercise practices, with less attention towards evaluating knowledge and attitudes. While the practice domain is well-researched, this restricts our understanding of the underlying mechanisms driving behavioral change. According to the Knowledge, Attitude, and Practice (KAP) model, knowledge and attitudes are foundational elements that influence behavior [20]. Having a better understanding of knowledge and attitudes regarding diet and exercise practices can provide valuable insights into strategies for promoting behavioral change [219]. This information can be used to tailor interventions to address specific areas where cancer survivors lack confidence in their ability to make healthier lifestyle choices, promote self-management, and improve healthcare provider practices [219,220]. Hence, addressing this gap could provide deeper insights into effective strategies for promoting sustainable behavioral change that could lead to improved desirable outcomes.

There appears to be increasing interest in research around NE-KAP (in particular practice domains), given its direct and measurable connection to health outcomes in cancer survivorship over the years. However, the diversity of questionnaires used remains high, with a lack of standardization or prior studies to validate them in a certain population prior to their use, i.e., content, face, and construct validity [221]. A previous systematic review of the GS-LTPAQ in oncology research revealed that while the questionnaire was originally designed for ranking or classification (i.e., level of total physical activity; active or insufficiently active) purposes, other studies have since modified it for other reasons such as screening for or evaluation of intervention. This extended scope of use of the GS-LTPAQ or any other questionnaires highlights the need for researchers in oncology to standardize its uses and interpretation in the cancer population [222].

Therefore, it may be more scientifically sound to first standardize the use of these questionnaires and develop guidelines for adaptations to certain populations before delving further into this area of research. This approach aligns with recommendations from recent studies, which emphasize the need for standardized tools to improve the reliability and comparability of research findings in cancer survivorship [223]. Future research should prioritize the use of validated and standardized assessment questionnaires to enhance the quality and reliability of findings in the field of survivorship care. The results from this review suggest that the adaptations of questionnaires prior to a study may not be regulated or explicitly directed by guidelines, relying heavily on justifications (if any) provided by authors for each modification. The standardization of these questionnaires and/or provision of a guide for adaptation would address heterogeneity, allow for more precise comparisons across studies, and enhance the synthesis of evidence.

In addition, current research focusing on the knowledge and attitude domain is limited due to variability in how concepts like ‘exercise knowledge’ or ‘attitudes toward healthy eating’ are defined across studies, leading to inconsistencies in measurement and interpretation. Moreover, the absence of uniformity hinders the ability to draw reliable conclusions or develop evidence-based interventions in this domain. Several studies indicate a more complex relationship between knowledge, attitude, and practice [224,225]. There is no doubt that knowledge and attitudes play a significant role in mediating behavioral change, not only among cancer survivors but also among service professionals within health systems [43,226,227]. While most assessment questionnaires identified in the current review align with the practice domain, there is a pressing need to expand research on knowledge and attitude assessment questionnaires. Understanding the mechanisms underlying behavioral change requires a deeper exploration of how knowledge and attitudes influence health-related practices. By incorporating these domains into research, we can develop more effective, targeted interventions that address not only what individuals do but also why they do it, ultimately leading to more sustainable behavioral changes and improved health outcomes for cancer survivors.

When carefully selected or adapted, results from these NE-KAP assessment questionnaires can provide valuable insights into cancer survivors’ understanding, motivation, and behavioral patterns around the cornerstones of lifestyle changes, i.e., diet and physical activity. These insights can directly inform the design of survivorship care interventions (e.g., tailored education programs or referral to nutrition–exercise support services) to bridge gaps in knowledge and encourage sustained healthy practices [228,229]. In clinical settings, incorporating NE-KAP assessments into routine follow-up can help healthcare providers proactively address barriers to lifestyle changes through education/counselling or by referring on to a nutrition or exercise specialist [230,231].

Although a comprehensive search was conducted in line with the agreed scope of work (i.e., covering a 5-year period), relevant studies published before this timeframe were not captured. Thus, the present study can only offer a snapshot of contemporary trends in NE-KAP within cancer survivorship research. As only studies in English were included, assessment questionnaires used from non-English sources were not included. This brings to light another significant issue regarding the implementation of these tools/questionnaires globally. Factors outside of predominantly English-speaking countries, such as different cultures, religions, and languages, can significantly influence the effectiveness and accessibility of cancer survivorship programs. For instance, two studies by Kasherman et al. highlighted the disparities faced by culturally and linguistically diverse (CALD) communities in accessing cancer care and survivorship programs and emphasizes the importance of culturally sensitive and specific assessments and interventions to ensure optimal care for survivors from diverse backgrounds [232,233].

## 5. Conclusions

Many NE-KAP assessment questionnaires are used in research involving cancer survivors. Around one-quarter of questionnaires were adapted to specific studies with no or unreported psychometric evaluation of questionnaires to the cancer population prior to use. Emphasis was placed on assessing nutrition and exercise practices, with limited attention towards knowledge (e.g., food label reading, exercise intensity) and attitude (e.g., attitude towards fruits and vegetables, intention to be physically active) domains. Future research should prioritize the standardization of NE-KAP assessment questionnaires or explore the validity of these existing questionnaires in diverse populations within the cancer survivorship cohort, particularly those underrepresented in research, such as CALD and Indigenous communities.

## Figures and Tables

**Table 1:** List of inclusion and exclusion criteria for scoping review.

Inclusion Criteria	Exclusion Criteria
Observational (e.g., cross-sectional, case-control) and interventional studies (e.g., randomized controlled trials); Mixed studies that include both quantitative and qualitative components in English	Reviews (including systematic reviews), qualitative-only studies, or studies not in English
Adult cancer survivors (18 years or older)	Children, young adults < 18 years old; animals, in vivo, in vitro.
Definition of cancer survivor from Clinica Oncology of Society of Australia—from diagnosis for the remainder of their life [34]	Non-cancer survivor population if not mentioned explicitly, and in less than 25% of the study population.
Studies that report nutrition and/or exercise knowledge, attitude, and/or practices.	If knowledge or attitude pertaining to cancer diagnosis only, and does not include either nutrition or exercise knowledge, attitudes, or practice; e.g., screening practices for breast cancer recurrence; assessment questionnaires requiring open-ended (text) answers (e.g., how do you feel about exercise?)

**Table 2:** Directory of nutrition–exercise knowledge, attitude, practice (NE-KAP) assessment questionnaires (*n* = 200) from 262 studies, by domain and frequency of use in cancer survivorship research.

Nutrition	*n*	Exercise	*n*
**Knowledge**			
2015 French Cancer Barometer Survey	1	2015 French Cancer Barometer Survey	1
6-Item Study Specific Questionnaire [42]	1	Exercise Processes of Change Questionnaire	1
Health Education Impact Questionnaire (heiQ)	1	Health Education Impact Questionnaire (heiQ)	1
* Study Specific Questionnaire [41]	1	Health Promotion Lifestyle Profile II (HPLP-II) PA Subscale	1
		Planning, Attitudes, and Barriers (PAB) Scale	1
		Related to Others Physical Activity (ROPAS)	1
		Theory of Planned Behavior (TPB) Questionnaire [43]	1
		* Study Specific Questionnaires [39,40,44,45]	4
**Attitude**			
10-Item Questionnaire Adapted from the Reasons for Quitting (Intrinsic and Extrinsic Motivation) scale	1	16-Item Decisional Balance	1
2015 French Cancer Barometer Survey	1	2015 French Cancer Barometer Survey	1
Acceptance and Action Questionnaire-II (AAQ-II)	1	9-Item Multidimensional Self-efficacy for Exercise Scale (MSES) [46,47,48]	3
Eating Beliefs Questionnaire (EBQ-18)	1	Abbreviated Self Leadership Questionnaire (ASLQ)	1
Health Education Impact Questionnaire (heiQ)	1	Acceptance and Action Questionnaire-II (AAQ-II)	1
Mindful Eating Questionnaire (MEQ)	1	Barriers Specific Self-Efficacy Scale (BARSE)	1
Modified Food Beliefs Survey	1	Basic Psychological Needs in Exercise Scale (BPNES)	1
National Cancer Institute’s Food Attitudes and Behaviors (FAB) Survey	1	Behavioral Regulation in Exercise Questionnaire (BREQ)	1
Nutrition Self-Efficacy Scale	1	Brief Physical Activity-Related Psychosocial Measures	1
Perceived Access to Healthy Foods survey	1	18-Item Exercise Self-Efficacy Questionnaire [49]	1
Physical Activity and Nutrition Self-Efficacy (PANSE)	1	Decisional Balance Scale	1
Preferences and Self-Efficacy of Diet and Physical Activity Behaviors Questionnaire for Latina Women (PSEDPALW)	1	Exercise Barriers/Benefits Scale (EBBS)	1
Social Support for Healthy Eating Questionnaire	1	Exercise Importance Scale [44]	1
The 14-item scale: Confidence Preparing a Variety of Plant Foods	1	Exercise Self-Efficacy Questionnaire [50]	1
The 8-item Perceived Health Competence Scale	1	Exercise Self-Efficacy Scale (ESES)	4
The Short Form of the Food-Life Questionnaire [FLQ-SF]	1	Five-Factor Model Questionnaire	1
Three-Factor Eating Questionnaire (TFEQ)	1	General Self-Efficacy Scale (Sherer’s)	1
Weight Efficacy Lifestyle (WEL) Questionnaire	1	Health Action Process Approach (HAPA) Constructs Questionnaire [51]	1
* Study Specific questionnaires [41,52,53,54,55]	5	Health Education Impact Questionnaire (heiQ)	1
		Inventory of Motivation to the Regular Practice of Physical Activity	1
		Lifestyle Efficacy Scale	1
		Mobility-Related Self-Efficacy (MRSE)	1
		Multidimensional Outcome Expectations for Exercise Scale	2
		Neighborhood Environment Walkability Scale (NEWS): Short Form	2
		New General Self-Efficacy scale	1
		Physical Activity Acceptance Questionnaire (PAAQ)	1
		Population-Based Questionnaire (modified using TPB)	1
		Preferences and Self-Efficacy of Diet and Physical Activity Behaviors Questionnaire for Latina Women (PSEDPALW)	1
		Psychometric Assessment of Action and Coping Planning [56]	1
		Psychosocial Questionnaire [57]	1
		Self-Efficacy and Stages of Exercise Behavior Change Questionnaire	1
		Self-Efficacy for Exercise Behaviors Scale	1
		Self-Efficacy to Perform Self-Management Behaviors Questionnaire	1
		Social Support for Exercise Survey	1
		Spanish Self-Efficacy Scale for Physical Activity (EAF)	1
		Spanish version of the Exercise Benefits/Barriers Scale	1
		Survey of Exercise Barrier Scale	1
		TPB questionnaire [18,58]	2
		* Study Specific Questionnaire [38,39,40,45,51,54,55,59,60,61,62,63,64,65,66,67]	16
**Practice**			
120-Item Women’s Health Initiative FFQ [68]	1	15-Item scale (American Cancer Society’s Cancer Prevention Study)	1
2015 French Cancer Barometer Survey	1	2015 French Cancer Barometer Survey	1
204-Item semi-quantitative FFQ (adapted from Division of Human Nutrition and Health, Wagenin University and Research 104-Item FFQ) [69]	2	Active Australia survey	1
23-Item Brief Dietary Assessment Questionnaire for Hispanics	1	Australian Longitudinal Study on Women’s Health (ALSWH) survey	3
30-Item Dietary Screener Questionnaire (DSQ)	2	Arizona Activity Frequency Questionnaire (AAFQ)	2
5-A-Day Measure Questionnaire	1	Automated Heart-Health Assessment Questionnaire	1
Acceptance and Action Questionnaire-II (AAQ-II)	1	Baecke Questionnaire	2
Adapted AUDIT-C Questionnaire	1	Basic Psychological Needs in Exercise Scale (BPNES)	1
Arizona FFQ (AFFQ)	1	Behavioral Regulation in Exercise Questionnaire (BREQ)	4
ALSWH Survey (Dietary Questionnaire for Epidemiology studies)	3	Behavioral Risk Factor Surveillance Survey (BRFSS)	8
Automated Heart-Health Assessment Questionnaire	1	Brief Physical Assessment Questionnaire	1
Automated Self-Administered 24-h (ASA24)	2	Community Health Activities Model Program for Seniors (CHAMPS) Questionnaire	5
Behavioral Risk Factor Surveillance Survey (BRFSS)	8	European Prospective Investigation into Cancer and Nutrition Study (EPIC) Physical Activity Questionnaire	2
Block 2005 FFQ	4	Epi-GEICAM Study Survey	1
Block Fruit/ Vegetable/Fiber and Fat Intake Screeners	1	Exercise Processes of Change Questionnaire	1
Canadian Adaptation of the US National Cancer Institute (NCI)’s Past Year Diet History Questionnaire II (CDHQ-II)	1	Exercise Vital Sign	1
City of Hope Quality of Life–Ostomy (COH-QOL-O) Survey	1	French vie après le Cancer (VICAN) Survey	1
Diet History Questionnaire II (DHQ II)	2	Frequency of Food Consumption of Adolescents and Adults Survey	2
Dietary Instrument for Nutrition Education (DINE) for Snacks	1	GEM Study Lifestyle Questionnaire (adapted from the Paffenbarger Physical Activity Questionnaire (PPAQ))	1
Dietary Questionnaire for Epidemiological Studies (DQES) Version 2	1	Global Physical Activity Questionnaire (GPAQ)	14
Digital DIGIKOST-FFQ	1	Godin–Shephard Leisure–Time Physical Activity Questionnaire (GSLTPAQ)	52
Eating at America’s Table Study Quick Food Scan	1	HAPA Constructs	1
European Prospective Investigation into Cancer and Nutrition Norfolk FFQ	1	Health Coaching Questionnaire [70]	1
Food-Based Lifelines Diet Score (LLDS)	1	NCI Health Information National Trends Survey (HINTS)	3
Frequency of Food Consumption of Adolescents and Adults Survey	2	Health-Promoting Lifestyle Profile II (HPLP-II)	3
Health Promotion Lifestyle Profile II (HPLP-II) Nutrition Subscale	1	International North Eastern German Society of Gynecological Oncology (NOGGO), European Network of Gynaecological Oncological Trial Groups (ENGOT), and Gynecologic Cancer InterGroup (GCIG) Survey	1
Healthy Lifestyle Instrument for Breast Cancer Survivors	1	International Physical Activity Questionnaire (IPAQ)	26
International NOGGO, ENGOT, and GCIG Survey	1	Korean National Health and Nutrition Examination Question Set on Aerobic Exercise	3
Japanese version of the 10-Item Eating Assessment Questionnaire (EAT-10)	1	Kriska and Caspersen (Global Physical Activity Question (GPAq) Questionnaire	1
Korean National Health and Nutrition Examination Survey on Diet Practices	1	Lifetime Total Physical Activity Questionnaire (LTPAQ)	1
Lymphedema Self-Management Behavior Questionnaire for Breast Cancer (LSMBQ-BC)	1	Lymphedema Self-Management Behavior Questionnaire for Breast Cancer (LSMBQ-BC)	1
MedDiet Questionnaire	1	Medical Outcomes Study 36-Item Short Form Health Survey (MOS-SF-36)	1
National Institutes of Health (NIH) Fruit and Vegetable Intake Screeners in the Eating at America’s Table Study (NIHEATS) ALL Day Questionnaire	1	Minnesota Questionnaire on Physical Activities, Sports, and Leisure Brazil version	1
National Health and Nutrition Examination Survey (NHANES)	2	Modifiable Activity Questionnaire (MAQ)	1
National Health Interview Survey (NHIS)	1	National Health and Nutrition Examination Survey (NHANES)	2
NCI Health Information National Trends Survey (HINTS)	3	National Health Interview Survey (NHIS)	1
NCI Dietary Screener Questionnaire (DSQ)	2	National Physical Activity Guidelines for Australian Adults	1
NCI FAB Survey (Fruit and Vegetable, Fat Screener)	1	Nord-Trondelag Health Study Physical Activity Questionnaire (HUNT 1PA-Q)	1
NCI Eating at America’s Table Screener (EATS) FFQ	5	Past Year Total Physical Activity Questionnaire (PYTPAQ)	1
NCI Multifactor Screener	1	Patient-Reported Charlson Comorbidity Index (PRO-CCI) Questionnaire	1
Rapid Eating Assessment for Participants—Short (REAP-S)	2	Personal Habits Questionnaire (Form 35) from Women’s Health Initiative Clinical Trial and Observational Study [71]	1
Self-Report SCREEN II	1	Physical Activity Group Environment Questionnaire (PAGE-Q)	1
Spices and Herbs Questionnaire [72]	1	Physical Activity Habits (Level and Frequency): Linear Analog Scale Assessment Items	1
Starting the Conversation Diet	2	Physical Activity Scale for the Elderly	1
Three-Factor Eating Questionnaire (TFEQ)	1	Physical Component Summary (PCS) of the 12-Item Short-Form Health Survey [73]	1
VioScreen 30-day FFQ	3	Rapid Assessment Physical Activity Scale (RAPA)	1
Women’s Health Initiative FFQ	1	Reproductive Window in Young Adult Cancer Survivors (Window) Study Survey	1
* Study Specific Questionnaire [74,75,76,77,78,79,80,81,82,83,84,85,86,87]	14	Self-Administered Physical Activity Questionnaire (PAQ)	1
		Short Questionnaire to Assess Health-Enhancing Physical Activity (SQUASH)	9
		Southern Community Cohort Study Baseline Questionnaire (Physical Activity)	1
		Stanford 7-Day Physical Activity Recall (7-Day PAR)	1
		Stanford Leisure–Time Activity Categorical Item (L-CAT)	1
		Stanford Patient Education Research Center Exercise Behaviors Survey	1
		The Accountable Health Communities Health-Related Social Needs Screening Questionnaire	1
		World Health Organization (WHO) STEPwise Approach to Noncommunicable Diseases Risk Factor Surveillance (STEPS)	2
		Women’s Health Initiative Brief Physical Activity Questionnaire (WHI-BPAQ)	1
		* Study Specific Questionnaires [38,39,41,51,74,76,77,79,82,84,86,88,89,90,91,92,93,94,95,96,97,98]	22

* Study specific questionnaires are untitled questionnaires developed by the lead author(s) as referenced. For titled questionnaires with generic names that may be difficult to locate, references were provided. N, number of included studies that used this assessment questionnaire.

## Data Availability

The original contributions presented in the study are included in the article/Supplementary Material, further inquiries can be directed to the corresponding author.

## Supplementary Materials

The following supporting information can be downloaded at: https://www.mdpi.com/article/10.3390/nu17091412/s1, Table S1: PRISMA-ScR checklist; Table S2: Search strategy; File S1: Full reference list of included studies (*n* = 262).
