# Supplementary material for: Nutrition and Exercise Knowledge, Attitude, and Practice: A Scoping Review of Assessment Questionnaires in Cancer Survivorship
Source: Nutrients. 2025 Apr 23;17(9):1412. doi: 10.3390/nu17091412 (PMC12073881; doi:10.3390/nu17091412)
Supplement: Supplementary file 1 [file nutrients-17-01412-s001.zip › Supplementary materials File S1 - list of included studies_262.pdf]

Table S3. List of included studies (n=262)

1. Acito M, Rondini T, Gargano G, Moretti M, Villarini M, Villarini A. How the COVID-19 pandemic has affected eating habits and physical activity in breast cancer survivors: the DianaWeb study. Journal of Cancer Survivorship. 2023;17(4):974-85.
2. Adams L, Feike J, Eckert T, Gohner W, Sporhase U, Bitzer E-M. Effectiveness of a motivational-volitional group intervention to increase physical activity among breast cancer survivors compared to standard medical rehabilitation-Study protocol of a prospective controlled bi-centred interventional trial. European journal of cancer care. 2019;28(4):e13073.
3. Alexander GK, Bashore L, Brooks V. Improving Food Literacy and Access among Young Adult Cancer Survivors: A Cross-Sectional Descriptive Study. Cancer nursing. 2022;45(2):161-6.
4. An K-Y, Kang D-W, Courneya KS. Correlates of Aerobic and Strength Exercise in Korean Cancer Patients: Data From the 2014–2016 Korea National Health and Nutrition Examination Survey. Cancer nursing. 2022;45(1):E255-E62.
5. An K-Y, Kang D-W, Morielli AR, Friedenreich CM, Reid RD, McKenzie DC, et al. Patterns and predictors of exercise behavior during 24 months of follow-up after a supervised exercise program during breast cancer chemotherapy. International Journal of Behavioral Nutrition & Physical Activity. 2020;17(1):1-11.
6. An K-Y, Morielli AR, Kang D-W, Friedenreich CM, McKenzie DC, Gelmon K, et al. Effects of exercise dose and type during breast cancer chemotherapy on longer-term patient-reported outcomes and health-related fitness: A randomized controlled trial. International journal of cancer. 2020;146(1):150-60.
7. Asare M, McIntosh S, Culakova E, Alio A, Umstattd Meyer MR, Kleckner AS, et al. Assessing Physical Activity Behavior of Cancer Survivors by Race and Social Determinants of Health. International quarterly of community health education. 2019;40(1):7-16.
8. Atoui S, Bernard P, Carli F, Liberman AS. Association Between Physical Activity, Sedentary Behaviors, and Sleep-Related Outcomes Among Cancer Survivors: a Cross-Sectional Study. International Journal of Behavioral Medicine. 2023;01:01.
9. Bail JR, Bail SV, Cagle J, Tiesi K, Caffey J, Bakitas M, et al. Health behaviors and well-being among those "living" with metastatic cancer in Alabama. Supportive Care in Cancer. 2022;30(2):1689-701.
10. Bail JR, Blair CK, Smith KP, Oster RA, Kaur H, Locher JL, et al. Harvest for Health, a Randomized Controlled Trial Testing a Home-Based, Vegetable Gardening Intervention Among Older Cancer Survivors Across Alabama: An Analysis of Accrual and Modifications Made in Intervention Delivery and Assessment During COVID-19. Journal of the Academy of Nutrition & Dietetics. 2022;122(9):1629-43.
11. Bao Y, Chen S, Jiang R, Li Y, Chen L, Li F, et al. The physical activity of colorectal cancer survivors during chemotherapy : Based on the theory of planned behavior. Supportive Care in Cancer. 2019;27(6):N.PAG-N.PAG.
12. Bastas D, Tabaczynski A, Whitehorn A, Trinh L. Preferences and engagement with physical activity resources among cancer survivors during the COVID-19 pandemic. Supportive Care in Cancer. 2023;31(7):374.
13. Beebe-Dimmer JL, Ruterbusch JJ, Harper FWK, Baird TM, Finlay DG, Rundle AG, et al. Physical activity and quality of life in African American cancer survivors: The Detroit Research on Cancer Survivors study. Cancer (0008543X). 2020;126(1):1987-94.

14. Bergengren O, Enblad AP, Garmo H, Bratt O, Holmberg L, Johansson E, et al. Changes in lifestyle among prostate cancer survivors: A nationwide population-based study. Psycho-oncology. 2020;29(10):1713-9.
15. Berzins NJ, Mackenzie M, Galantino ML, Pickles N, Hebbel S, Leonard T, et al. Preliminary Effectiveness Study of a Community-Based Wellness Coaching for Cancer Survivors Program. American Journal of Lifestyle Medicine. 2022.
16. Beverly Hery CM, Janse SA, Van Zee KJ, Naftalis EZ, Paskett ED, Naughton MJ. Factors associated with insomnia symptoms over three years among premenopausal women with breast cancer. Breast Cancer Research & Treatment. 2023;202(1):155-65.
17. Biskup M, Macek P, Terek-Derszniak M, Zak M, Krol H, Falana K, et al. Agreement between Accelerometer-Assessed and Self-Reported Physical Activity and Sedentary Behavior in Female Breast Cancer Survivors. Diagnostics. 2023;13(22):15.
18. Blair CK, McDougall JA, Chiu VK, Wiggins CL, Rajput A, Harding EM, et al. Correlates of poor adherence to a healthy lifestyle among a diverse group of colorectal cancer survivors. Cancer causes & control : CCC. 2019;30(12):1327-39.
19. Bluethmann SM, Foo W, Winkels RM, Mama SK, Schmitz KH. Physical Activity in Older Cancer Survivors: What Role Do Multimorbidity and Perceived Disability Play? Journal of Aging & Physical Activity. 2020;28(2):311-9.
20. Bluethmann SM, Keadle SK, King TS, Matthews CE, Perna FM. Rethinking physical activity assessment in cancer survivors: a multi-component approach using NHANES data. Journal of cancer survivorship : research and practice. 2022;16(4):781-90.
21. Bluethmann SM, Wang M, Wasserman E, Chen C, Zaorsky NG, Hohl RJ, et al. Prostate cancer in Pennsylvania: The role of older age at diagnosis, aggressiveness, and environmental risk factors on treatment and mortality using data from the Pennsylvania Cancer Registry. Cancer medicine. 2020;9(10):3623-33.
22. Bohn S, Oldervoll LM, Reinertsen KV, Seland M, Fossa A, Kiserud C, et al. The feasibility of a multidimensional intervention in lymphoma survivors with chronic fatigue. Supportive Care in Cancer. 2023;32(1):22.
23. Boing L, Fretta TB, Lynch BM, Dias M, Rosa LMD, Baptista F, et al. Mat Pilates and belly dance: Effects on patient-reported outcomes among breast cancer survivors receiving hormone therapy and adherence to exercise. Complementary Therapies in Clinical Practice. 2023;50:101683.
24. Boyd P, Lowry M, Morris KL, Land SR, Agurs-Collins T, Hall K, et al. Health Behaviors of Cancer Survivors and Population Controls From the National Health Interview Survey (2005-2015). JNCI cancer spectrum. 2020;4(5):pkaa043.
25. Brown NI, Pekmezi DW, Oster RA, Courneya KS, McAuley E, Ehlers DK, et al. Relationships between Obesity, Exercise Preferences, and Related Social Cognitive Theory Variables among Breast Cancer Survivors. Nutrients. 2023;15(5):1286.
26. Bruneau M, Jr., Milliron B-J, Sinclair E, Obeid E, Gross L, Bealin L, et al. Physical activity assessment among men undergoing genetic counseling for inherited prostate cancer: a teachable moment for improved survivorship. Supportive care in cancer : official journal of the Multinational Association of Supportive Care in Cancer. 2021;29(4):2145-51.
27. Brunet J, Howell D, Au D, Jones JM, Bradley H, Berlingeri A, et al. Predictors of cancer survivors' response to a community-based exercise program. Psychology of Sport & Exercise. 2020;47:N.PAG-N.PAG.
28. Buro AW, Carson TL, Small BJ, Fan W, Oswald LB, Jim HSL, et al. Sociocultural factors associated with physical activity in Black prostate cancer survivors. Supportive Care in Cancer. 2023;31(8):482.

29. Buro AW, Stern M, Carson TL. Reported Mental Health, Diet, and Physical Activity in Young Adult Cancer Survivors. Nutrients. 2023;15(4):1005.
30. Burse NR, Weng X, Wang L, Cuffee YL, Veldheer S. Influence of social and behavioral determinants on health-related quality of life among cancer survivors in the USA. Supportive Care in Cancer. 2023;31(1):67.
31. Buscemi J, Oswald LB, Baik SH, Buitrago D, Iacobelli F, Phillips SM, et al. My health smartphone intervention decreases daily fat sources among Latina breast cancer survivors. Journal of behavioral medicine. 2020;43(5):732-42.
32. Cao C, Friedenreich CM, Yang L. Association of Daily Sitting Time and Leisure-Time Physical Activity With Survival Among US Cancer Survivors. JAMA oncology. 2022;8(3):395-403.
33. Caperchione CM, Sabiston CM, Stolp S, Bottorff JL, Campbell KL, Eves ND, et al. A preliminary trial examining a 'real world' approach for increasing physical activity among breast cancer survivors: findings from project MOVE. BMC cancer. 2019;19(1):1-12.
34. Caperchione CM, Stolp S, Phillips JL, Agar M, Sharp P, Liauw W, et al. Cancer survivors' exercise beliefs, knowledge, and behaviors: An Australian National Survey. Asia-Pacific journal of clinical oncology. 2022;18(6):625-33.
35. Carmack CL, Parker NH, Demark-Wahnefried W, Shely L, Baum G, Yuan Y, et al. Healthy Moves to Improve Lifestyle Behaviors of Cancer Survivors and Their Spouses: Feasibility and Preliminary Results of Intervention Efficacy. Nutrients. 2021;13(12):4460-.
36. Carraca EV, Rodrigues B, Franco S, Nobre I, Jeronimo F, Ilharco V, et al. Promoting physical activity through supervised vs motivational behavior change interventions in breast cancer survivors on aromatase inhibitors (PAC-WOMAN): protocol for a 3-arm pragmatic randomized controlled trial. BMC Cancer. 2023;23(1):632.
37. Chakraborty R, Yi J, Rybicki L, Preussler J, Deol A, Loren A, et al. Patient-Reported Outcomes in Long-Term Survivors of Autologous Hematopoietic Cell Transplantation in Multiple Myeloma. Transplantation and Cellular Therapy. 2023;29(6):388.e1-.e6.
38. Choi S, Park NJ, Kim M, Song K, Choi J. Comparison of cardiovascular disease risk in women with and without breast cancer: secondary data analysis with the 2014-2018 korean national health and nutrition examination survey. BMC Public Health. 2023;23(1):1158.
39. Chung J, Kulkarni GS, Bender J, Breau RH, Guttman D, Maganti M, et al. Modifiable lifestyle behaviours impact the health-related quality of life of bladder cancer survivors. BJU international. 2020;125(6):836-42.
40. Cole A, Andrilla CHA, Patterson D, Davidson S, Mendoza J. Measuring the Impact of the COVID-19 Pandemic on Health Behaviors and Health Care Utilization in Rural and Urban Patients with Cancer and Cancer Survivors. Cancer research communications. 2023;3(2):215-22.
41. Coletta AM, Marquez G, Thomas P, Thoman W, Bevers T, Brewster AM, et al. Clinical factors associated with adherence to aerobic and resistance physical activity guidelines among cancer prevention patients and survivors. PloS one. 2019;14(8):e0220814.
42. Connor AE, Dibble KE, Visvanathan K. Lifestyle factors in Black female breast cancer survivors-Descriptive results from an online pilot study. Frontiers in Public Health. 2023;11:1072741.
43. Conway RE, Rigler FV, Croker HA, Lally PJ, Beeken RJ, Fisher A. Dietary supplement use by individuals living with and beyond breast, prostate, and colorectal cancer: A cross-sectional survey. Cancer. 2022;128(6):1331-8.
44. Cortés-Ibáñez FO, Jaramillo-Calle DA, Vinke PC, Byambasukh O, Corpeleijn E, Sijtsma A, et al. Comparison of health behaviours between cancer survivors and the general population: a

cross-sectional analysis of the Lifelines cohort. Journal of Cancer Survivorship. 2020;14(3):377-85.
45. Coughlin SS, Datta B, Majeed B. Preventive Behaviors Among Leukemia and Lymphoma Cancer Survivors: Results From the 2020 Behavioral Risk Factor Surveillance System Survey. AJPM focus. 2023;2(1).
46. Coughlin SS, Datta B, Moore JX, Vernon MM, Tingen MS. Preventive behaviors and behavioral risk factors among gynecologic cancer survivors: Results from the 2020 Behavioral Risk Factor Surveillance System Survey. Cancer Medicine. 2023;12(14):15435-46.
47. Cox-Martin E, Cox MG, Basen-Engquist K, Bradley C, Blalock JA. Changing multiple health behaviors in cancer survivors: smoking and exercise. Psychology, health & medicine. 2020;25(3):331-43.
48. Cuesta-Vargas AI, Biro A, Escriche-Escuder A, Trinidad-Fernandez M, Garcia-Conejo C, Roldan Jimenez CR, et al. Effectiveness of a gamified digital intervention based on lifestyle modification (iGAME) in secondary prevention: a protocol for a randomised controlled trial. BMJ open. 2023;13(6):e066669.
49. de Lima Melo B, Vieira DCA, de Oliveira GC, Valente J, Sanchez Z, Ferrari G, et al. Adherence to healthy lifestyle recommendations in Brazilian cancer survivors. Journal of Cancer Survivorship. 2023;17(6):1751-9.
50. De Liz S, Vieira FGK, Geraldo APG, de Assis MAA, Di Pietro PF. Educational nutritional intervention program improved the quality of diet of women with breast cancer in adjuvant treatment. Revista de Nutricao. 2020;33:1-15.
51. Demark-Wahnefried W, Oster RA, Crane TE, Rogers LQ, Cole WW, Kaur H, et al. Results of DUET: A Web-Based Weight Loss Randomized Controlled Feasibility Trial among Cancer Survivors and Their Chosen Partners. Cancers. 2023;15(5):03.
52. Deng F, Chen D, Swartz MC, Sun H. A Pilot Study of a Culturally Tailored Lifestyle Intervention for Chinese American Cancer Survivors. Cancer Control: Journal of the Moffitt Cancer Center. 2019;26:N.PAG-N.PAG.
53. Di Meglio A, Martin E, Crane TE, Charles C, Barbier A, Raynard B, et al. A phase III randomized trial of weight loss to reduce cancer-related fatigue among overweight and obese breast cancer patients: MEDEA Study design. Trials. 2022;23(1):1-16.
54. Dibble KE, Connor AE. Evaluation of disparities in maintaining healthy lifestyle behaviors among female cancer survivors by race/ethnicity and US nativity. Cancer epidemiology. 2022;80:102235.
55. Din HN, Strong D, Singh-Carlson S, Corliss HL, Hartman SJ, Madanat H, et al. Association between pregnancy intention and preconception health behaviors. Cancer. 2022;128(3):615-23.
56. Doyle C, Ko E, Lemus H, Hsu FC, Pierce JP, Wu T. Living Alone, Physical Health, and Mortality in Breast Cancer Survivors: A Prospective Observational Cohort Study. Healthcare. 2023;11(17):24.
57. Driessen KAJ, de Rooij BH, Vos MC, Boll D, Pijnenborg JMA, Hoedjes M, et al. Cancer-related psychosocial factors and self-reported changes in lifestyle among gynecological cancer survivors: cross-sectional analysis of PROFILES registry data. Supportive Care in Cancer. 2022;30(2):1199-207.
58. Duchek D, McDonough MH, Bridel W, McNeely ML, Culos-Reed SN. Understanding In-Person and Online Exercise Oncology Programme Delivery: A Mixed-Methods Approach to Participant Perspectives. Current Oncology. 2023;30(8):7366-83.
59. Edmonds MC, Bickell NA, Gallagher EJ, LeRoith D, Lin JJ. Racial differences in weight perception among Black and White women diagnosed with breast cancer. Journal of Cancer Survivorship. 2022.

60. Ehlers DK, Fanning J, Sunderlage A, Severson J, Kramer AF, McAuley E. Influence of sitting behaviors on sleep disturbance and memory impairment in breast cancer survivors. Cancer medicine. 2020;9(10):3417-24.
61. Entwistle MR, Schweizer D, Cisneros R. Adherence to dietary patterns among cancer survivors in the United States. Journal of Public Health (09431853). 2022;30(7):1651-60.
62. Ergas IJ, Bradshaw PT, Cespedes Feliciano EM, Roh JM, Kwan ML, Laraia B, et al. Hypothetical Interventions on Diet Quality and Lifestyle Factors to Improve Breast Cancer Survival: The Pathways Study. Cancer Epidemiology, Biomarkers & Prevention. 2023;32(12):1716-25.
63. Evans C, Saliba-Serre B, Préau M, Bendiane M-K, Gonçalves A, Signoli M, et al. Post-traumatic growth 5 years after cancer: identification of associated actionable factors. Supportive Care in Cancer. 2022;30(10):8261-70.
64. Fallon EA, Stephens RL, McDonald B, Diefenbach M, Leach CR. Disentangling Efficacy and Expectations: A Prospective, Cross-lagged Panel Study of Cancer Survivors' Physical Activity. Annals of Behavioral Medicine. 2019;53(2):138-49.
65. Friedenreich CM, Vallance JK, McNeely ML, Culos-Reed SN, Matthews CE, Bell GJ, et al. The Alberta moving beyond breast cancer (AMBER) cohort study: baseline description of the full cohort. Cancer causes & control : CCC. 2022;33(3):441-53.
66. Fu Y, Li K, Zhou Z, Wei W, Wang C, Dong J, et al. Integrating Self-determination Theory and Upper Limb Factors to Predict Physical Activity in Patients With Breast Cancer During Chemotherapy. Cancer nursing. 2022;45(1):52-60.
67. Gjerset GM, Kiserud CE, Loge JH, Fosså SD, Wisløff T, Gudbergsson SB, et al. Changes in fatigue, health-related quality of life and physical activity after a one-week educational program for cancer survivors. Acta Oncologica. 2019;58(5):682-9.
68. Glasgow TE, McGuire KP, Fuemmeler BF. Eat, sleep, play: health behaviors and their association with psychological health among cancer survivors in a nationally representative sample. BMC cancer. 2022;22(1):1-11.
69. Golsteijn RHJ, Bolman C, Peels DA, Volders E, de Vries H, Lechner L. Long-term efficacy of a computer-tailored physical activity intervention for prostate and colorectal cancer patients and survivors: A randomized controlled trial. Journal of Sport & Health Science. 2023;12(6):690-704.
70. Gray MS, Judd SE, Sloane R, Snyder DC, Miller PE, Demark-Wahnefried W. Rural-urban differences in health behaviors and outcomes among older, overweight, long-term cancer survivors in the RENEW randomized control trial. Cancer Causes & Control. 2019;30(4):301-9.
71. Greenberg AL, Tolstykh IV, Van Loon K, Laffan A, Stanfield D, Steiding P, et al. Association between adherence to the American Cancer Society Nutrition and Physical Activity Guidelines and stool frequency among colon cancer survivors: a cohort study. Journal of cancer survivorship : research and practice. 2023;17(3):836-47.
72. Gregory K, Zhao L, Felder TM, Clay-Gilmour A, Eberth JM, Murphy EA, et al. Prevalence of health behaviors among cancer survivors in the United States. Journal of Cancer Survivorship. 2023.
73. Gu Q, Dummer TBJ, Spinelli JJ, Murphy RA. Diet Quality among Cancer Survivors and Participants without Cancer: A Population-Based, Cross-Sectional Study in the Atlantic Partnership for Tomorrow's Health Project. Nutrients. 2019;11(12):3027-.
74. Gunn KM, Berry NM, Meng X, Wilson CJ, Dollman J, Woodman RJ, Clark RA, Koczwara B. Differences in the health, mental health and health-promoting behaviours of rural versus urban cancer survivors in Australia. Support Care Cancer. 2020 Feb;28(2):633-643.

75. Haas ND, Viele C, Paul SM, Abrams G, Smoot B, Melisko M, et al. Polymorphisms in Cytokine Receptor and Regulator Genes are Associated with Levels of Exercise in Women Prior to Breast Cancer Surgery. Biological Research for Nursing. 2023;25(1):76-87.
76. Hamed Bieyabanie M, Mirghafourvand M. Health Promoting Lifestyle and its Relationship with Self-Efficacy in Iranian Mastectomized Women. Asian Pacific journal of cancer prevention : APJCP. 2020;21(6):1667-72.
77. Han CJ, Tounkara F, Kalady M, Noonan AM, Burse NR, Paskett ED, et al. Risk Factors of Health-Related Quality of Life among Gastrointestinal Cancer Survivors in the U.S.: With a Focus on Social and Behavioral Determinants of Health (SBDH). International Journal of Environmental Research & Public Health [Electronic Resource]. 2023;20(17):29.
78. Han J, Jang MK, Lee H, Kim SY, Kim SH, Hee Ko Y, et al. Long Term Effects of a Social Capital-Based Exercise Adherence Intervention for Breast Cancer Survivors With Moderate Fatigue: A Randomized Controlled Trial. Integrative Cancer Therapies. 2023;22:15347354231209440.
79. Haynam ML, Chaplow ZL, DeScenza VR, Bowman JD, Dispennette K, Zhang X, et al. Design and methods of a translational, community-based, lifestyle weight management pilot intervention trial in breast cancer survivors with overweight or obesity. Contemporary Clinical Trials Communications. 2023;33:101154.
80. Hiensch AE, Peeters PHM, Jansen M, Wall EVD, Backx FJG, Velthuis MJ, et al. Socio-ecological correlates of physical activity in breast and colon cancer survivors 4 years after participation in a randomized controlled exercise trial (PACT study). PloS one. 2020;15(4).
81. Hill EB, Grainger EM, Young GS, Clinton SK, Spees CK. Application of the Updated WCRF/AICR Cancer Prevention Score as an Outcome for Cancer Survivors Participating in a Tailored and Intensive Dietary and Physical Activity Intervention. Nutrients. 2022;14(22):4751.
82. Hoang T, Lee J, Kim J, Park B. Food Intake Behavior in Cancer Survivors in Comparison With Healthy General Population; From the Health Examination Center-based Cohort. Journal of cancer prevention. 2019;24(4):208-16.
83. Huang S, Riccardi D, Pflanzer S, Redwine LS, Gray HL, Carson TL, et al. Survivors Overcoming and Achieving Resiliency (SOAR): Mindful Eating Practice for Breast Cancer Survivors in a Virtual Teaching Kitchen. Nutrients. 2023;15(19):29.
84. Ichijo Y, Takeda Y, Oguma Y, Kitagawa Y, Takeuchi H, Doorenbos AZ. Physical Activity Among Postoperative Esophageal Cancer Patients. Cancer nursing. 2019;42(6):501-8.
85. Jeong JR, Choe YR. Health-promoting behaviors among middle-aged breast cancer survivors compared with matched non-cancer controls: A KNHANES VI-VII (2013-2018) study. Medicine. 2023;102(26):e34065.
86. Joaquim A, Amarelo A, Antunes P, Garcia C, Leão I, Vilela E, et al. Effects of a Physical Exercise Program on Quality of Life and Physical Fitness of Breast Cancer Survivors: the MAMA_MOVE Gaia After Treatment Trial. Psychology, Health and Medicine. 2023.
87. Johnson RS, Fallon EA, Berg CJ. Correlates of light physical activity among cancer survivors. Psycho-oncology. 2019;28(4):726-34.
88. Jones TL, Edbrooke L, Rawstorn JC, Hayes SC, Maddison R, Denehy L, et al. Self-efficacy, motivation, and habits: psychological correlates of exercise among women with breast cancer. Supportive Care in Cancer. 2023;31(10):584.
89. Jun HS, Lee K. Association Between Fear of Cancer Recurrence, Fatigue, and Healthy Lifestyle Behaviors Among Breast Cancer Survivors in South Korea. Cancer nursing. 2023.

90. Kang D-Q, Li Y, Chen Z-Q, Liu Q, Su C-X, Guo H, et al. Correlates of Physical Activity in Colorectal Cancer Patients Based on Health Promotion Model. Cancer nursing. 2020;43(5):E264-E72.
91. Kaur H, Pavela G, Pekmezi DW, Rogers LQ, Cole WW, Parrish KB, et al. Dietary Barriers Appear to Influence the Effects of a Dyadic Web-Based Lifestyle Intervention on Caloric Intake and Adiposity: A Mediation Analysis of the DUET Trial. Nutrients. 2023;15(23):25.
92. Keaver L, O'Callaghan N, Douglas P. Nutrition support and intervention preferences of cancer survivors. J Hum Nutr Diet. 2023 Apr;36(2):526-539.
93. Kellogg Parsons JK, Zahrieh D, Mohler JL, Paskett E, Hansel DE, Kibel AS, et al. Effect of a Behavioral Intervention to Increase Vegetable Consumption on Cancer Progression among Men with Early-Stage Prostate Cancer: The MEAL Randomized Clinical Trial. JAMA - Journal of the American Medical Association. 2020;323(2):140-8.
94. Kelly DL, Yang GS, Starkweather AR, Siangphoe U, Alexander-Delpech P, Lyon DE. Relationships Among Fatigue, Anxiety, Depression, and Pain and Health-Promoting Lifestyle Behaviors in Women With Early-Stage Breast Cancer. Cancer nursing. 2020;43(2):134-46.
95. Kendall SJ, Heinze S, Blanchard C, Chiekwe JC, Melvin J, Culos-Reed N, et al. Exercise Programming Modelling a Standard of Care Approach Improves Physical Health and Patient-Reported Outcomes in Individuals Living with Breast Cancer: A Pilot Study. Current Oncology. 2023;30(8):7203-17.
96. Kenfield SA, Philip EJ, Phillips SM, Meyerhardt JA, Chan JM, Atreya CE, et al. Optimizing intervention tools to improve nutrition and physical activity for colorectal cancer survivors (Tools To Be Fit): Study protocol of a randomized factorial experiment. Contemporary clinical trials. 2022;123:107009.
97. Kenkhuis M-F, Mols F, van Roekel EH, Breedveld-Peters JJL, Breukink SO, Janssen-Heijnen MLG, et al. Longitudinal Associations of Adherence to the World Cancer Research Fund/American Institute for Cancer Research (WCRF/AICR) Lifestyle Recommendations with Quality of Life and Symptoms in Colorectal Cancer Survivors up to 24 Months Post-Treatment. Cancers. 2022;14(2):417-.
98. Kenkhuis MF, Klingestijn M, Fanshawe AM, Breukink SO, Janssen-Heijnen MLG, Keulen ETP, et al. Longitudinal associations of sedentary behavior and physical activity with body composition in colorectal cancer survivors up to 2 years post treatment. Journal of Cancer Research & Clinical Oncology. 2023;149(7):4063-75.
99. Kennedy F, Lally P, Miller NE, Conway RE, Roberts A, Croker H, et al. Fatigue, quality of life and associations with adherence to the World Cancer Research Fund guidelines for health behaviours in 5835 adults living with and beyond breast, prostate and colorectal cancer in England: A cross-sectional study. Cancer Medicine. 2023;12(11):12705-16.
100. Kim I, Lim JY, Kim SW, Shin DW, Kim HC, Park YA, et al. Effectiveness of personalized treatment stage-adjusted digital therapeutics in colorectal cancer: a randomized controlled trial. BMC Cancer. 2023;23(1):304.
101. Kim J, Kang S, Kim D, Kang H. Associations of Physical Activity and Handgrip Strength with Health-Related Quality of Life in Older Korean Cancer Survivors. Cancers. 2022;14(24):6067.
102. Kim J, Keegan TH. Characterizing risky alcohol use, cigarette smoking, e-cigarette use, and physical inactivity among cancer survivors in the USA-a cross-sectional study. Journal of Cancer Survivorship. 2023;17(6):1799-812.
103. Kim K, Xu W, Hong SJ, Starkweather A, Brown RF, Walsh S. Perceived discrimination and physical activity mediate the associations between receiving a survivorship care plan and cancer pain. Cancer epidemiology. 2022;78:102155.

104. Kim Y, Franco RL, Lucas AR, Sutton AL, LaRose JG, Kenyon J, et al. Prevalence of Cardiovascular Diseases Among Breast Cancer Survivors: Findings From the NHANES 2003-2018. American Journal of Health Promotion. 2023;37(2):233-8.
105. Kindred MM, Pinto BM, Dunsiger SI. Predictors of sedentary behavior among colorectal survivors. Supportive Care in Cancer. 2019;27(6):2049-56.
106. Kiplagat K, Antoine F, Ramos R, Nahid M, Forte V, Taiwo E, et al. An Acceptance Based Lifestyle Intervention in Black Breast Cancer Survivors with Obesity. Journal of immigrant and minority health. 2021.
107. Kirkham AA, Pituskin E, Neil-Sztramko SE. Age-dependent increased odds of cardiovascular risk factors in cancer survivors: Canadian Longitudinal Study on Aging cohort. Current Oncology. 2020;27(4):e368-e76.
108. Koch PA, Paul R, Contento IR, Gray HL, Marin-Chollom AM, Santiago-Torres M, et al. Mi Vida Saludable: Content Validity and Reliability of The Preferences and Self-Efficacy of Diet and Physical Activity Behaviors Questionnaire for Latina Women (PSEDPALW) for Cancer Survivors. Nutrients. 2023;15(16):12.
109. Kotte M, Bolam KA, Mijwel S, Altena R, Cormie P, Wengstrom Y. Distance-based delivery of exercise for people treated for breast, prostate or colorectal cancer: a study protocol for a randomised controlled trial of EX-MED Cancer Sweden. Trials. 2023;24(1):116.
110. Koutoukidis DA, Beeken RJ, Manchanda R, Burnell M, Ziauddeen N, Michalopoulou M, et al. Diet, physical activity, and health-related outcomes of endometrial cancer survivors in a behavioral lifestyle program: the Diet and Exercise in Uterine Cancer Survivors (DEUS) parallel randomized controlled pilot trial. International journal of gynecological cancer : official journal of the International Gynecological Cancer Society. 2019;29(3):531-40.
111. Krok-Schoen JL, Pisegna J, Arthur E, Ridgway E, Stephens C, Rosko AE. Prevalence of lifestyle behaviors and associations with health-related quality of life among older female cancer survivors. Supportive Care in Cancer. 2021;29(6):3049-59.
112. Kwan ML, Valice E, Ergas IJ, Roh JM, Caan BJ, Cespedes Feliciano EM, et al. Alcohol consumption and prognosis and survival in breast cancer survivors: The Pathways Study. Cancer. 2023;129(24):3938-51.
113. Kwarteng JL, Matthews L, Banerjee A, Sharp LK, Gerber BS, Stolley MR. The association of stressful life events on weight loss efforts among African American breast cancer survivors. Journal of Cancer Survivorship. 2021.
114. Lambert SD, Duncan LR, Ellis J, Robinson JW, Sears C, Culos-Reed N, et al. A study protocol for a multicenter randomized pilot trial of a dyadic, tailored, web-based, psychosocial, and physical activity self-management program (TEMPO) for men with prostate cancer and their caregivers. Pilot and feasibility studies. 2021;7(1):78.
115. Langlais CS, Graff RE, Van Blarigan EL, Neuhaus JM, Cowan JE, Broering JM, et al. Post-diagnostic health behaviour scores and risk of prostate cancer progression and mortality. British Journal of Cancer. 2023;129(2):346-55.
116. Le Y, Gao Z, Gomez SL, Pope Z, Dong R, Allen L, et al. Acculturation and Adherence to Physical Activity Recommendations Among Chinese American and Non-Hispanic White Breast Cancer Survivors. Journal of Immigrant & Minority Health. 2019;21(1):80-8.
117. Leach HJ, Covington KR, Voss C, LeBreton KA, Harden SM, Schuster SR. Effect of Group Dynamics- Based Exercise Versus Personal Training in Breast Cancer Survivors. Oncology nursing forum. 2019;46(2):185-97.
118. Leach HJ, Crisafio ME, Howell MJ, Nicklawsky A, Marker RJ. A Group-Based, Videoconference-Delivered Physical Activity Program for Cancer Survivors. Translational Journal of the American College of Sports Medicine. 2023;8(2).

119. Lee E, Zhu J, Velazquez J, Bernardo R, Garcia J, Rovito M, et al. Evaluation of Diet Quality Among American Adult Cancer Survivors: Results From 2005-2016 National Health and Nutrition Examination Survey. Journal of the Academy of Nutrition & Dietetics. 2021;121(2):217-32.
120. Lee J, Min J, Lee DH, Kang D-W, Jeon JY. Intensity- and domain-specific physical activity levels between cancer survivors and non-cancer diagnosis individuals: a propensity score matching analysis. Supportive Care in Cancer. 2021;29(2):661-8.
121. Lee JS, Park M, Kim YH. Sedentary Behavior and Physical Activity of Community-Dwelling Korean Breast Cancer Survivors: A Nationwide Study. Healthcare. 2023;11(13):07.
122. Lee MK. Decisional balance, self-leadership, self-efficacy, planning, and stages of change in adopting exercise behaviors in patients with stomach cancer: A cross-sectional study. European Journal of Oncology Nursing. 2022;56:N.PAG-N.PAG.
123. Lesser I, Janzen A, Arshad N, Wurz A. Describing and exploring physical activity experiences among adults with cancer during the COVID-19 pandemic: a mixed-methods survey study. Journal of psychosocial oncology. 2023:1-9.
124. Lesser IA, Nienhuis CP, Belanger L. Active by nature: exploring cancer survivors' exercise barriers, facilitators, preferences, and psychosocial benefits of engaging in outdoor physical activity. Supportive Care in Cancer. 2021;29(7):4095-103.
125. Lévesque-Gagné C, Boucher D. Psychosocial factors associated with the intention of breast cancer survivors to regularly practise moderate physical activity. Canadian Oncology Nursing Journal. 2022;32(3):348-56.
126. Liao Y, Schembre SM, Brannon GE, Pan Z, Wang J, Ali S, et al. Using wearable biological sensors to provide personalized feedback to motivate behavioral changes: Study protocol for a randomized controlled physical activity intervention in cancer survivors (Project KNOWN). PloS one. 2022;17(9):e0274492.
127. Liao Y, Song J, Robertson MC, Cox-Martin E, Basen-Engquist K. An Ecological Momentary Assessment Study Investigating Self-efficacy and Outcome Expectancy as Mediators of Affective and Physiological Responses and Exercise Among Endometrial Cancer Survivors. Annals of Behavioral Medicine. 2020;54(5):320-34.
128. Ligibel JA, Zheng Y, Barry WT, Sella T, Ruddy KJ, Greaney ML, et al. Effects of an educational physical activity intervention in young women with newly diagnosed breast cancer: Findings from the Young and Strong Study. Cancer. 2023.
129. Lin AW, Marchese SH, Finch LE, Stump T, Gavin KL, Spring B. Obesity Status on associations between cancer-related beliefs and health behaviors in cancer survivors: Implications for patient-clinician communication. Patient Education & Counseling. 2021;104(8):2067-72.
130. Lindgren A, Dunberger G, Steineck G, Bergmark K, Enblom A. Identifying female pelvic cancer survivors with low levels of physical activity after radiotherapy: women with fecal and urinary leakage need additional support. Supportive Care in Cancer. 2020;28(6):2669-81.
131. Liu SY, Lu L, Pringle D, Mahler M, Niu C, Charow R, et al. Impact of immigration status on health behaviors and perceptions in cancer survivors. Cancer medicine. 2019;8(5):2623-35.
132. Liu VN, Zuniga KB, Paciorek A, Zhang L, Chan JM, Carroll PR, et al. Barriers and confidence among colorectal and prostate cancer survivors participating in two behavioral intervention studies. Supportive Care in Cancer. 2023;31(8):453.
133. Lope V, Guerrero-Zotano A, Ruiz-Moreno E, Bermejo B, Antolín S, Montaño Á, et al. Clinical and Sociodemographic Determinants of Adherence to World Cancer Research Fund/American Institute for Cancer Research (WCRF/AICR) Recommendations in Breast Cancer Survivors—Health-EpiGEICAM Study. Cancers. 2022;14(19):4705.

134. Lozano-Lozano M, Cantarero-Villanueva I, Martin-Martin L, Galiano-Castillo N, Sanchez M-J, Fernandez-Lao C, et al. A Mobile System to Improve Quality of Life Via Energy Balance in Breast Cancer Survivors (BENECA mHealth): Prospective Test-Retest Quasiexperimental Feasibility Study. JMIR mHealth and uHealth. 2019;7(6):e14136.
135. Lucas AR, Kim Y, Lanoye A, Franco RL, Sutton AL, LaRose JG, et al. Longitudinal associations among physical activity and sitting with endocrine symptoms and quality of life in breast cancer survivors: A latent growth curve analysis. Cancer Medicine. 2023;12(19):20094-105.
136. Lucas AR, Pan JH, Ip EH, Hall DL, Tooze JA, Levine B, et al. Validation of the Lee-Jones theoretical model of fear of cancer recurrence among breast cancer survivors using a structural equation modeling approach. Psycho-Oncology. 2023;32(2):256-65.
137. Mama SK, Bhuiyan N, Foo W, Segel JE, Bluethmann SM, Winkels RM, et al. Rural-urban differences in meeting physical activity recommendations and health status in cancer survivors in central Pennsylvania. Supportive Care in Cancer. 2020;28(10):5013-22.
138. Mama SK, Bhuiyan N, Smyth JM, Schmitz KH. Stress and Physical Activity in Rural Cancer Survivors: The Moderating Role of Social Support. Journal of Rural Health. 2020;36(4):543-8.
139. Mama SK, Heredia NI, Johnston H, Conroy DE. Associations Between Physical Activity and Alcohol Consumption in Rural Cancer Survivors. Frontiers in Oncology. 2022;12:871192.
140. Manne S, Devine K, Hudson S, Kashy D, O'Malley D, Paddock LE, et al. Factors associated with health-related quality of life in a cohort of cancer survivors in New Jersey. BMC Cancer. 2023;23(1):664.
141. Maras J, Murray AB, Boardley D, Van Wasshenova E, Tull MT, Tipton J, et al. Assessing the Relationship Between Implicit and Explicit Evaluations of Fruit and Vegetable Consumption by Cancer Survivors. International journal of behavioral medicine. 2019;26(4):365-71.
142. Marell PS, Vierkant RA, Olson JE, Herrmann J, Larson N, LeBrasseur NK, et al. Factors Associated With Physical Activity Levels in Patients With Breast Cancer. Oncologist. 2022;27(10):e811-e4.
143. Martin-Nunez J, Linares-Moya M, Calvache-Mateo A, Lazo-Prados A, Heredia-Ciuro A, Lopez-Lopez L, et al. Barriers and applied activity, quality of life and self-efficacy in prostate cancer survivors 1 year after completing radiotherapy. Supportive Care in Cancer. 2023;31(5):284.
144. Maxwell-Smith C, Hagger MS, Kane R, Cohen PA, Tan J, Platell C, et al. Psychological correlates of physical activity and exercise preferences in metropolitan and nonmetropolitan cancer survivors. Psycho-oncology. 2021;30(2):221-30.
145. Mbous YPV, Mohamed R, Bhandari R. A Decomposition Analysis of Racial Disparities in Physical Activity Among Cancer Survivors: National Health Interview Survey 2009-2018. Journal of physical activity & health. 2023:1-12.
146. McGinnis EL, Rogers LQ, Fruhauf CA, Jankowski CM, Crisafio ME, Leach HJ. Feasibility of Implementing Physical Activity Behavior Change Counseling in an Existing Cancer-Exercise Program. International journal of environmental research and public health. 2021;18(23).
147. McMenamin E, Gottschalk AB, Pucci DA, Jacobs LA. Health behaviors among head and neck cancer survivors. Journal of health, population, and nutrition. 2023;42(1):48.
148. Millar MM, Herget KA, Ofori-Atta B, Codden RR, Edwards SL, Carter ME, et al. Cancer survivorship experiences in Utah: an evaluation assessing indicators of survivors' quality of life, health behaviors, and access to health services. Cancer causes & control : CCC. 2023;34(4):337-47.

149. Miller MF, Li Z, Habedank M. A Randomized Controlled Trial Testing the Effectiveness of Coping with Cancer in the Kitchen, a Nutrition Education Program for Cancer Survivors. Nutrients. 2020;12(10):3144.
150. Min J, Yoo S, Kim M-J, Yang E, Hwang S, Kang M, et al. Exercise participation, barriers, and preferences in Korean prostate cancer survivors. Ethnicity & health. 2021;26(8):1130-42.
151. Min J, Yu Y-W, Lee J, Yeon S, Park H-N, Lee JS, et al. Application of the theory of planned behavior to understand physical activity intentions and behavior among Korean breast cancer survivors. Supportive Care in Cancer. 2022;30(11):8885-93.
152. Mizrahi D, Goldstein D, Trinh T, Li T, Timmins HC, Harrison M, et al. Physical activity behaviors in cancer survivors treated with neurotoxic chemotherapy. Asia-Pacific journal of clinical oncology. 2023;19(1):243-9.
153. Mo J, Thomson CA, Sun V, Wendel CS, Hornbrook MC, Weinstein RS, et al. Healthy behaviors are associated with positive outcomes for cancer survivors with ostomies: a cross-sectional study. Journal of Cancer Survivorship. 2021;15(3):461-9.
154. Moraitis AM, Rose NB, Johnson AF, Dunston ER, Garrido-Laguna I, Hobson P, et al. Feasibility and acceptability of an mHealth, home-based exercise intervention in colorectal cancer survivors: A pilot randomized controlled trial. PLoS ONE [Electronic Resource]. 2023;18(6):e0287152.
155. Murray AB, Boardley D, Wasshenova EV, Mahas R, Kiviniemi MT, Tipton J, et al. Affective determinants of physical activity in cancer survivors. Psychology & health. 2020;35(5):593-612.
156. Nakandi K, Benebo FO, Hopstock LA, Stub T, Kristoffersen AE. Adherence to lifestyle recommendations among Norwegian cancer survivors and the impact of traditional and complementary medicine use: the Tromsø Study 2015–2016. BMC Complementary Medicine & Therapies. 2023;23(1):1-12.
157. Natalucci V, Ferri Marini C, De Santi M, Annibalini G, Lucertini F, Vallorani L, et al. Movement and health beyond care, MoviS: study protocol for a randomized clinical trial on nutrition and exercise educational programs for breast cancer survivors. Trials. 2023;24(1):1-15.
158. Nechuta SJ, Lipworth L, Chen WY, Shu XO, Zheng W, Blot WJ. Physical activity in association with mortality among Black women diagnosed with breast cancer in the Southern Community Cohort Study. Cancer causes & control : CCC. 2023;34(3):277-86.
159. Nejati B, Lin C-Y, Griffiths M, Pakpour A. Psychometric Properties of the Persian Food-Life Questionnaire Short Form among Obese Breast Cancer Survivors. Asia-Pacific Journal of Oncology Nursing. 2020;7(1):64-71.
160. Nelson D, McGonagle I, Jackson C, Tsuro T, Scott E, Gussy M, et al. Health-Promoting Behaviours following Primary Treatment for Cancer: A Rural-Urban Comparison from a Cross-Sectional Study. Current Oncology. 2023;30(2):1585-97.
161. Ng AH, Ngo-Huang A, Vidal M, Reyes-Garcia A, Liu DD, Williams JL, et al. Exercise Barriers and Adherence to Recommendations in Patients With Cancer. JCO oncology practice. 2021;17(7):e972-e81.
162. Olson JL, Robertson M, Chen M, Conroy DE, Schmitz KH, Mama SK. Healthier Energy Balance Behaviors Most Important for Health-Related Quality of Life in Rural Cancer Survivors in Central Pennsylvania. Journal of physical activity & health. 2023:1-8.
163. Onyeaka HK, Zambrano J, Longley RM, Celano CM, Naslund JA, Amonoo HL. Use of digital health tools for health promotion in cancer survivors. Psycho-oncology. 2021;30(8):1302-10.

164. Packel L, Dychtwald D, Pontiggia L, Deutsch J, Milliron BJ. Physical Activity and Nutrition-Related Beliefs, Behaviors, and Challenges in Individuals Living with Cancer and Their Caregivers. Rehabilitation Oncology. 2023;41(1):23-33.
165. Page LL, Kahn CJ, Severson J, Kramer AF, McAuley E, Ehlers DK. Physical activity and cognitive function: A comparison of rural and urban breast cancer survivors. PloS one. 2023;18(4 April):e0284189.
166. Papadopoulos E, Leach HJ, Tomlinson G, Durbano S, Danyluk JM, Sabiston CM, et al. Factors predicting gains in moderate-to-vigorous physical activity in prostate cancer survivors on androgen deprivation therapy. Supportive Care in Cancer. 2022;30(11):9011-8.
167. Park J, Kim J, Shin DW, Shin J, Cho B, Song YM. Factors Associated with Dietary Habit Changes in Korean Stomach Cancer Survivors after Cancer Treatment. Nutrients. 2023;15(14):24.
168. Park S-H. Health-Promoting Lifestyle Behaviors of Korean American Breast Cancer Survivors: Adherence to the American Cancer Society Guidelines. Cancer nursing. 2022.
169. Park S-H, Knobf MT, Kerstetter J, Jeon S. Adherence to American Cancer Society Guidelines on Nutrition and Physical Activity in Female Cancer Survivors: Results From a Randomized Controlled Trial (Yale Fitness Intervention Trial). Cancer nursing. 2019;42(3):242-50.
170. Park S-H, Strauss SM. Correlates of Physical Health Comorbidities Among Adult Female Cancer Survivors in South Korea. Oncology nursing forum. 2022;49(6):585-94.
171. Parker NH, Basen-Engquist K, Rubin ML, Li Y, Prakash L, Ngo-Huang A, et al. Factors Influencing Exercise Following Pancreatic Tumor Resection. Annals of surgical oncology. 2021;28(4):2299-309.
172. Paunescu AC, Preau M, Jacob G, Pannard M, Delrieu L, Delpierre C, et al. Health behaviour changes in female cancer survivors: The Seintinelles study. Bulletin du Cancer. 2023;110(5):496-511.
173. Pekmezi D, Fontaine K, Rogers LQ, Pisu M, Martin MY, Schoenberger-Godwin Y-M, et al. Adapting MultiPLe behavior Interventions that eFfectively Improve (AMPLIFI) cancer survivor health: program project protocols for remote lifestyle intervention and assessment in 3 inter-related randomized controlled trials among survivors of obesity-related cancers. BMC cancer. 2022;22(1):1-15.
174. Pinto BM, Dunsiger SI, DeScenza VR, Stein K. Mediators of physical activity outcomes in a peer-led intervention for breast cancer survivors. Psycho-Oncology. 2023;32(4):619-27.
175. Pinto BM, Dunsiger SI, Kindred MM, Mitchell S. Mediators of physical activity maintenance during a 12-month randomized controlled trial among breast Cancer survivors. Journal of Behavioral Medicine. 2023;46(5):745-56.
176. Pisegna J, Xu M, Spees C, Krok-Schoen JL. Mental health-related quality of life is associated with diet quality among survivors of breast cancer. Supportive Care in Cancer. 2021;29(4):2021-8.
177. Pophali PA, Larson MC, Rosenthal AC, Robinson D, Habermann TM, Thanarajasingam G, et al. The association of health behaviors with quality of life in lymphoma survivors. Leukemia & lymphoma. 2021;62(2):271-80.
178. Porter KJ, Moon KE, LeBaron VT, Zoellner JM. A Novel Behavioral Intervention for Rural Appalachian Cancer Survivors (weSurvive): Participatory Development and Proof-of-Concept Testing. JMIR Cancer. 2021;7(2):e26010.
179. Price J, Barrett-Bernstein M, Wurz A, Karvinen KH, Brunet J. Health beliefs and engagement in moderate-to-vigorous-intensity physical activity among cancer survivors: a cross-sectional study. Supportive Care in Cancer. 2021;29(1):477-84.

180. Pudkasam S, Pitcher M, Fisher M, O'Connor A, Chinlumprasert N, Stojanovska L, et al. The PAPHIO study protocol: a randomised controlled trial with a 2 x 2 crossover design of physical activity adherence, psychological health and immunological outcomes in breast cancer survivors. BMC public health. 2020;20(1):696.
181. Puklin LS, Harrigan M, Cartmel B, Sanft T, Gottlieb L, Zhou B, et al. Randomized Trial Evaluating a Self-Guided Lifestyle Intervention Delivered via Evidence-Based Materials versus a Waitlist Group on Changes in Body Weight, Diet Quality, Physical Activity, and Quality of Life among Breast Cancer Survivors. Cancers. 2023;15(19):25.
182. Rastogi S, Tevaarwerk AJ, Sesto M, Van Remortel B, Date P, Gangnon R, et al. Effect of a technology-supported physical activity intervention on health-related quality of life, sleep, and processes of behavior change in cancer survivors: A randomized controlled trial. Psycho-oncology. 2020;29(11):1917-26.
183. Revesz D, Bonhof CS, Bours MJL, Weijenberg MP, Vreugdenhil G, van de Poll-Franse LV, et al. Sociodemographic, Clinical, Lifestyle, and Psychological Correlates of Peripheral Neuropathy among 2- to 12-Year Colorectal Cancer Survivors. Oncology research and treatment. 2022;45(9):480-93.
184. Revesz D, Bours MJL, Wegdam JA, Keulen ETP, Breukink SO, Slooter GD, et al. Longitudinal associations of sociodemographic, lifestyle, and clinical factors with alcohol consumption in colorectal cancer survivors up to 2 years post-diagnosis. Supportive Care in Cancer. 2021;29(10):5935-43.
185. Riani Costa LA, F Barreto R, de Leandrini SMM, Gurgel ARB, de Sales GT, Voltarelli VA, et al. The influence of a supervised group exercise intervention combined with active lifestyle recommendations on breast cancer survivors' health, physical functioning, and quality of life indices: study protocol for a randomized and controlled trial. Trials. 2021;22(1):934.
186. Ricci C, Freisling H, Leitzmann MF, Taljaard-Krugell C, Jacobs I, Kruger HS, et al. Diet and sedentary behaviour in relation to cancer survival. A report from the national health and nutrition examination survey linked to the U.S. mortality registry. Clinical Nutrition. 2020;39(11):3489-96.
187. Robertson MC, Cox-Martin E, Shegog R, Markham CM, Fujimoto K, Durand CP, et al. The Acceptability of an Electronically Delivered Acceptance- and Mindfulness-Based Physical Activity Intervention for Survivors of Breast Cancer: One-Group Pretest-Posttest Design. JMIR Cancer. 2022;8(2):e31815.
188. Rogers LQ, Courneya KS, Oster RA, Anton PM, Phillips S, Ehlers DK, et al. Physical activity intervention benefits persist months post-intervention: randomized trial in breast cancer survivors. Journal of Cancer Survivorship. 2023.
189. Rubio MA, Mejia-Arbelaez CM, Wilches-Mogollon MA, Moreno S, Finck C, Rosas LG, et al. "My Body, My Rhythm, My Voice": a community dance pilot intervention engaging breast cancer survivors in physical activity in a middle-income country. Pilot & Feasibility Studies. 2023;9(1):30.
190. Ryu S, Adams K, Chen Y, Gao Z. Breast cancer survivors' physical activity, psychosocial beliefs, daily trip behaviors, and subjective well-being: A descriptive study. Complementary therapies in clinical practice. 2022;49:N.PAG-N.PAG.
191. Sabiston CM, Fong AJ, O'Loughlin EK, Meterissian S. A mixed-methods evaluation of a community physical activity program for breast cancer survivors. Journal of translational medicine. 2019;17(1):N.PAG-N.PAG.
192. Safdari-Molan M, Mehrabi E, Nourizadeh R, Eghdam-Zamiri R. Predictors of the worry about cancer recurrence among women with breast cancer. BMC women's health. 2023;23(1):131.

193. Salerno EA, Gao R, Fanning J, Gothe NP, Peterson LL, Anbari AB, et al. Designing home-based physical activity programs for rural cancer survivors: A survey of technology access and preferences. Frontiers in Oncology. 2023;13:1061641.
194. Samaroo K, Hosein A, Ali J. Perception of Survivorship Needs Among Breast Cancer Patients in Trinidad and Tobago. Cureus. 2023;15(1):e34394.
195. Sanft T, Harrigan M, Cartmel B, Ferrucci LM, Li F-Y, McGowan C, et al. Effect of healthy diet and exercise on chemotherapy completion rate in women with breast cancer: The Lifestyle, Exercise and Nutrition Early after Diagnosis (LEANer) study: Study protocol for a randomized clinical trial. Contemporary clinical trials. 2021;109:106508.
196. Schleicher E, McAuley E, Courneya KS, Anton P, Ehlers DK, Phillips SM, et al. Moderators of physical activity and quality of life response to a physical activity intervention for breast cancer survivors. Supportive Care in Cancer. 2023;31(1):53.
197. Sedano-Ochoa SA, Alvarez Banuelos MT, Saldana-Ibarra SA, Arroyo Helguera O, Coutino Rodriguez R. Sociodemographic Barriers to Physical Activity and Healthy Diet Through Social Networks in Mexican Breast Cancer Survivors. Cureus. 2023;15(10):e47678.
198. Seguin Leclair C, Lebel S, Westmaas JL. Can Physical Activity and Healthy Diet Help Long-Term Cancer Survivors Manage Their Fear of Recurrence? Frontiers in psychology. 2021;12:647432.
199. Sequeira M, Pereira C, Alvarez MJ. Predicting Physical Activity in Survivors of Breast Cancer: the Health Action Process Approach at the Intrapersonal Level. International Journal of Behavioral Medicine. 2023;30(6):777-89.
200. Shen A, Wu P, Qiang W, Fu X, Zhu F, Pang L, et al. Factors associated with lymphedema self-management behaviours among breast cancer survivors: A cross-sectional study. Journal of Clinical Nursing. 2023;32(19-20):7330-45.
201. Shi Z, Rundle A, Genkinger JM, Cheung YK, Ergas IJ, Roh JM, et al. Distinct trajectories of moderate to vigorous physical activity and sedentary behavior following a breast cancer diagnosis: the Pathways Study. Journal of Cancer Survivorship. 2020;14(3):393-403.
202. Shi Z, Rundle A, Genkinger JM, Cheung YK, Ergas IJ, Roh JM, et al. Distinct trajectories of fruits and vegetables, dietary fat, and alcohol intake following a breast cancer diagnosis: the Pathways Study. Breast cancer research and treatment. 2020;179(1):229-40.
203. Shimizu Y, Tsuji K, Ochi E, Arai H, Okubo R, Kuchiba A, et al. Study protocol for a nationwide questionnaire survey of physical activity among breast cancer survivors in Japan. BMJ open. 2020;10(1):032871.
204. Silva DTC, Vanderlei LCM, Palma MR, Ribeiro FE, Tebar WR, Tosello GT, et al. Association Between Different Domains of Physical Activity and Body Adiposity Indicators in Breast Cancer Survivors. Clinical breast cancer. 2022;22(4):e438-e43.
205. Singleton AC, Raeside R, Partridge SR, Hyun KK, Tat-Ko J, Sum SCM, et al. Supporting women's health outcomes after breast cancer treatment comparing a text message intervention to usual care: the EMPOWER-SMS randomised clinical trial. Journal of Cancer Survivorship. 2023;17(6):1533-45.
206. Skiba MB, Dieckmann NF, Lyons KS, Winters-Stone KM. Associations between perceptions of relationship quality and markers of inflammation and insulin resistance among couples coping with cancer. Journal of Cancer Survivorship. 2023;17(4):957-66.
207. Skiba MB, Jacobs ET, Crane TE, Kopp LM, Thomson CA. Relationship Between Individual Health Beliefs and Fruit and Vegetable Intake and Physical Activity Among Cancer Survivors: Results from the Health Information National Trends Survey. Journal of Adolescent & Young Adult Oncology. 2022;11(3):259-67.

208. Sohl SJ, Sadasivam RS, Kittel C, Dressler EV, Wentworth S, Balakrishnan K, et al. Pilot study of implementing the Shared Healthcare Actions & Reflections Electronic systems in Survivorship (SHARE-S) program in coordination with clinical care. Cancer medicine. 2023.
209. Soltero EG, James DL, Han S, Larkey LK. The impact of a meditative movement practice intervention on short- and long-term changes in physical activity among breast cancer survivors. Journal of Cancer Survivorship. 2023;28:28.
210. Song L, Guan T, Guo P, Tan X, Bryant AL, Wood WA, et al. Health behaviors, obesity, and marital status among cancer survivors: a MEPS study. Journal of Cancer Survivorship. 2023;17(2):499-508.
211. Spees CK, Braun AC, Hill EB, Grainger EM, Portner J, Young GS, et al. Impact of a Tailored Nutrition and Lifestyle Intervention for Overweight Cancer Survivors on Dietary Patterns, Physical Activity, Quality of Life, and Cardiometabolic Profiles. Journal of Oncology. 2019:1-13.
212. Springfield S, Odoms-Young A, Tussing-Humphreys L, Freels S, Stolley M. Adherence to American Cancer Society and American Institute of Cancer Research dietary guidelines in overweight African American breast cancer survivors. Journal of Cancer Survivorship. 2019;13(2):257-68.
213. Springfield S, Odoms-Young A, Tussing-Humphreys LM, Freels S, Stolley MR. A Step toward Understanding Diet Quality in Urban African-American Breast Cancer Survivors: A Cross-sectional Analysis of Baseline Data from the Moving Forward Study. Nutrition & Cancer. 2019;71(1):61-76.
214. St George SM, Noriega Esquives B, Agosto Y, Kobayashi M, Leite R, Vanegas D, et al. Development of a multigenerational digital lifestyle intervention for women cancer survivors and their families. Psycho-oncology. 2020;29(1):182-94.
215. Stan DL, Cutshall SM, Adams TF, Ghosh K, Clark MM, Wieneke KC, et al. Wellness Coaching: An Intervention to Increase Healthy Behavior in Breast Cancer Survivors. Clinical journal of oncology nursing. 2020;24(3):305-15.
216. Stolley MR, Sheean P, Matthews L, Banerjee A, Visotcky A, Papanek P, et al. Exploring health behaviors, quality of life, and support needs in African-American prostate cancer survivors: a pilot study to support future interventions. Supportive Care in Cancer. 2020;28(7):3135-43.
217. Stone CR, Courneya KS, McGregor SE, Li H, Friedenreich CM. Determinants of changes in physical activity from pre-diagnosis to post-diagnosis in a cohort of prostate cancer survivors. Supportive Care in Cancer. 2019;27(8):2819-28.
218. Sukumar JS, Vaughn JE, Tegge A, Sardesai S, Lustberg M, Stein J. Delay Discounting as a Potential Therapeutic Target for Weight Loss in Breast Cancer Survivors. Cancers. 2022;14(5):1134.
219. Sun V, Crane TE, Arnold KB, Guthrie K, Freylersythe S, Braun-Inglis C, et al. SWOG S1820: Altering Intake, Managing Symptoms for bowel dysfunction in survivors of Rectal Cancer (The AIMS-RC intervention trial). Contemporary clinical trials communications. 2021;22:100768.
220. Sweegers MG, Depenbusch J, Kampshoff CS, Aaronson NK, Hiensch A, Wengstrom Y, et al. Perspectives of patients with metastatic breast cancer on physical exercise programs: results from a survey in five European countries. Supportive Care in Cancer. 2023;31(12):694.
221. Tabaczynski A, Bastas D, Whitehorn A, Trinh L. Changes in physical activity and associations with quality of life among a global sample of cancer survivors during the COVID-19 pandemic. Journal of Cancer Survivorship. 2023;17(4):1191-201.

222. Tabaczynski A, Courneya KS, Trinh L. Replacing sedentary time with physical activity and sleep: associations with quality of life in kidney cancer survivors. Cancer Causes & Control. 2020;31(7):669-81.
223. Tami-Maury IM, Liao Y, Rangel ML, Gatus LA, Shinn EH, Alexander A, et al. Active Living After Cancer: Adaptation and evaluation of a community-based physical activity program for minority and medically underserved breast cancer survivors. Cancer. 2022;128(2):353-63.
224. Taschner MC, Piero R, Broomhall CN, Crecelius AR. Examining Contributors to Intent to Continue Exercising in Patients With Cancer in Rehabilitation. Clinical journal of oncology nursing. 2022;26(1):78-85.
225. Tavakol M, Ashtiani RT, Koosheshi M, Akbari ME, Khayamzadeh M. Social Disparities in Post-diagnosis Health Behaviors of Iranian Breast Cancer Survivors: The Mediating Role of Psychosocial Factors as Potential Buffering Agents. International Journal of Cancer Management. 2021;14(8):1-7.
226. Taylor KS, Beeken RJ, Fisher A, Lally P. Did the COVID-19 pandemic impact the dietary intake of individuals living with and beyond breast, prostate, and colorectal cancer and who were most likely to experience change? Supportive Care in Cancer. 2023;31(10):585.
227. Terranova CO, Winkler EAH, Healy GN, Demark-Wahnefried W, Eakin EG, Reeves MM. Dietary and Physical Activity Changes and Adherence to WCRF/AICR Cancer Prevention Recommendations following a Remotely Delivered Weight Loss Intervention for Female Breast Cancer Survivors: The Living Well after Breast Cancer Randomized Controlled Trial. Journal of the Academy of Nutrition & Dietetics. 2022;122(9):1644-.
228. Thomson CA, Crane TE, Miller A, Gold MA, Powell M, Bixel K, et al. Lifestyle intervention in ovarian cancer enhanced survival (LIVES) study (NRG/GOG0225): Recruitment, retention and baseline characteristics of a randomized trial of diet and physical activity in ovarian cancer survivors. Gynecologic oncology. 2023;170:11-8.
229. Tollosa DN, Holliday E, Hure A, Tavener M, James EL. Multiple health behaviors before and after a cancer diagnosis among women: A repeated cross-sectional analysis over 15 years. Cancer medicine. 2020;9(9):3224-33.
230. Tollosa DN, Holliday E, Hure A, Tavener M, James EL. A 15-year follow-up study on long-term adherence to health behaviour recommendations in women diagnosed with breast cancer. Breast cancer research and treatment. 2020;182(3):727-38.
231. Tollosa DN, Tavener M, Hure A, James EL. Compliance with Multiple Health Behaviour Recommendations: A Cross-Sectional Comparison between Female Cancer Survivors and Those with no Cancer History. International journal of environmental research and public health. 2019;16(8).
232. Tometich DB, Mosher CE, Cyders M, McDonald BC, Saykin AJ, Small BJ, et al. An Examination of the Longitudinal Relationship Between Cognitive Function and Physical Activity Among Older Breast Cancer Survivors in the Thinking and Living With Cancer Study. Annals of Behavioral Medicine. 2023;57(3):237-48.
233. Trinh L, Tabaczynski A, Bastas D, Neville AR, Voss ML, Whitehorn A. Changes in physical activity, sedentary behavior, and self-reported cognitive function in cancer survivors before and during the COVID-19 pandemic: A cross-sectional study. Journal of sport and health science. 2023.
234. Ueland K, Sanchez SC, Rillamas-Sun E, Shen H, Schattenkerk L, Garcia G, et al. A digital health intervention to improve nutrition and physical activity in breast cancer survivors: Rationale and design of the Cook and Move for Your Life pilot and feasibility randomized controlled trial. Contemporary clinical trials. 2022;123:106993.

235. Ulrich GR, Nogg KA, Freeman SZ, Ranby KW. Effects of remotely-delivered physical activity education on exercise beliefs and intentions of active and nonactive cancer survivors and their partners. Translational behavioral medicine. 2022;12(5):663-72.
236. Valle CG, Diamond MA, Heiling HM, Deal AM, Hales DP, Nezami BT, et al. Physical activity maintenance among young adult cancer survivors in an mHealth intervention: Twelve-month outcomes from the IMPACT randomized controlled trial. Cancer Medicine. 2023;12(15):16502-16.
237. Valle CG, Diamond MA, Heiling HM, Deal AM, Hales DP, Nezami BT, et al. Effect of an mHealth intervention on physical activity outcomes among young adult cancer survivors: The IMPACT randomized controlled trial. Cancer. 2023;129(3):461-72.
238. Vallerand JR, Rhodes RE, Walker GJ, Courneya KS. Social cognitive effects and mediators of a pilot telephone counseling intervention to increase aerobic exercise in hematologic cancer survivors. Journal of Physical Activity and Health. 2019;16(1):43-51.
239. Van Blarigan EL, Chan JM, Sanchez A, Zhang L, Winters-Stone K, Liu V, et al. Protocol for a 4-arm randomized controlled trial testing remotely delivered exercise-only, diet-only, and exercise + diet interventions among men with prostate cancer treated with radical prostatectomy (Prostate 8-II). Contemporary clinical trials. 2023;125:107079.
240. van Zutphen M, Boshuizen HC, Kok DE, van Baar H, Geijsen AJMR, Wesselink E, et al. Colorectal cancer survivors only marginally change their overall lifestyle in the first 2 years following diagnosis. Journal of Cancer Survivorship. 2019;13(6):956-67.
241. van Zutphen M, van Duijnhoven FJB, Wesselink E, Schrauwen RWM, Kouwenhoven EA, van Halteren HK, et al. Identification of Lifestyle Behaviors Associated with Recurrence and Survival in Colorectal Cancer Patients Using Random Survival Forests. Cancers. 2021;13(10).
242. Vd Wiel HJ, Stuiver MM, May AM, van Grinsven S, Benink MFA, Aaronson NK, et al. Characteristics of Participants and Nonparticipants in a Blended Internet-Based Physical Activity Trial for Breast and Prostate Cancer Survivors: Cross-sectional Study. JMIR Cancer. 2021;7(4):e25464.
243. Walsh JC, Richmond J, Mc Sharry J, Groarke A, Glynn L, Kelly MG, et al. Examining the Impact of an mHealth Behavior Change Intervention With a Brief In-Person Component for Cancer Survivors With Overweight or Obesity: Randomized Controlled Trial. JMIR mHealth and uHealth. 2021;9(7):e24915.
244. Waluya JG, Rahayuwati L, Lukman M. Supportive–educative nursing intervention on knowledge, attitude and physical activity intensity of survivors of breast cancer. Work. 2022;71(4):1137-44.
245. Wang TJ, Chang SC, Hsu HH, Huang CS, Lin TR, Lin YP, et al. Efficacy of a self-management program on quality of life in colorectal cancer patients: A randomized controlled trial. European Journal of Oncology Nursing. 2023;67:102431.
246. Weaver KE, Klepin HD, Wells BJ, Dressler EV, Winkfield KM, Lamar ZS, et al. Cardiovascular Assessment Tool for Breast Cancer Survivors and Oncology Providers: Usability Study. JMIR Cancer. 2021;7(1):e18396.
247. Wechsler S, Fu MR, Lyons K, Wood KC, Magee LJW. Role of Exercise Self-Efficacy in Exercise Participation Among Women With Persistent Fatigue After Breast Cancer: A Mixed-Methods Study. PTJ: Physical Therapy & Rehabilitation Journal. 2023;103(1):1-10.
248. Weller S, Oliffe JL, Campbell KL. Factors associated with exercise preferences, barriers and facilitators of prostate cancer survivors. European journal of cancer care. 2019;28(5):e13135.
249. Westrick AC, Langa KM, Kobayashi LC. The association of health behaviors prior to cancer diagnosis and functional aging trajectories after diagnosis: Longitudinal cohort study of middle-aged and older US cancer survivors. Preventive Medicine Reports. 2023;31:102083.

250. Williams V, Brown N, Moore JX, Farrell D, Perumean-Chaney S, Schleicher E, et al. Web-Based Lifestyle Interventions for Survivors of Cancer: Usability Study. JMIR formative research. 2022;6(2):e30974.
251. Wolff J, Wuelfing P, Koenig A, Ehrl B, Damsch J, Smollich M, et al. App-Based Lifestyle Coaching (PINK!) Accompanying Breast Cancer Patients and Survivors to Reduce Psychological Distress and Fatigue and Improve Physical Activity: A Feasibility Pilot Study. Breast Care. 2023;18(5):354-65.
252. Woopen H, Keller M, Zocholl D, Mittelstadt S, Barretina-Ginesta M-P, Heinzelmann-Schwarz V, et al. Side Effects from Cancer Therapies and Perspective of 1044 Long-Term Ovarian Cancer Survivors—Results of Expression VI–Carolin Meets HANNA–Holistic Analysis of Long-Term Survival with Ovarian Cancer: The International NOGGO, ENGOT, and GCIG Survey. Cancers. 2023;15(22):5428.
253. Yan R, Che B, Lv B, Wu P, Lu X, Zhang Y, et al. The association between physical activity, sedentary time and health-related quality of life in cancer survivors. Health & Quality of Life Outcomes. 2021;19(1):1-12.
254. Yin M, Wang C, Gu K, Bao P, Shu XO. Chronic pain and its correlates among long-term breast cancer survivors. Journal of Cancer Survivorship. 2023;17(2):460-7.
255. Youn J, Park S, Song S, Moon H-G, Noh D-Y, Jung S-Y, et al. Nutrient intakes from supplement and factors associated with supplement use among breast cancer survivors: A cross-sectional study. European journal of cancer care. 2021;30(5):e13447.
256. Yu CH, Wang TJ, Chang CL, Liang SY, Wu SF, Liu CY, et al. Healthy life styles, sleep and fatigue in endometrial cancer survivors: A cross-sectional study. Journal of Clinical Nursing (John Wiley & Sons, Inc). 2020;29(7/8):1372-80.
257. Yu Y, Cheng S, Huang H, Deng Y, Cai C, Gu M, et al. Joint association of sedentary behavior and vitamin D status with mortality among cancer survivors. BMC Medicine. 2023;21(1):411.
258. Yun YH, Lim CI, Lee ES, Kim YT, Shin KH, Kim YW, et al. Efficacy of health coaching and a web-based program on physical activity, weight, and distress management among cancer survivors: A multi-centered randomised controlled trial. Psycho-oncology. 2020;29(7):1105-14.
259. Zainordin NH, A Karim N, Shahril MR, Abd Talib R. Physical Activity, Sitting Time, and Quality of Life among Breast and Gynaecology Cancer Survivors. Asian Pacific journal of cancer prevention : APJCP. 2021;22(8):2399-408.
260. Zhang D, Feng Y, Li N, Sun X. Fruit and vegetable consumptions in relation to frequent mental distress in breast cancer survivors. Supportive Care in Cancer. 2021;29(1):193-201.
261. Zhu C, Lian Z, Chen Y, Wang J. Physical Activity and Cancer Status Among Middle-Aged and Older Chinese: A Population-Based, Cross-Sectional Study. Frontiers in physiology. 2021;12:812290.
262. Zuniga KE, Parma DL, Munoz E, Spaniol M, Wargovich M, Ramirez AG. Dietary intervention among breast cancer survivors increased adherence to a Mediterranean-style, anti-inflammatory dietary pattern: the Rx for Better Breast Health Randomized Controlled Trial. Breast cancer research and treatment. 2019;173(1):145-54.
