# Supplementary material for: Nutrition and Exercise Knowledge, Attitude, and Practice: A Scoping Review of Assessment Questionnaires in Cancer Survivorship
Source: Nutrients. 2025 Apr 23;17(9):1412. doi: 10.3390/nu17091412 (PMC12073881; doi:10.3390/nu17091412)
Supplement: Supplementary file 1 [file nutrients-17-01412-s001.zip › Supplementary materials Table S2 - Medline Search Strategy.pdf]

Search strategy after consultation with librarian – NE-KAP scoping review

DATABASE: Medline

#	Searches
1	Cancer Survivors/
2	exp neoplasms/
3	(cancer* or tumor* or tumour* or neoplas* or malignan* or carcinoma* or adenocarcinoma* or choriocarcinoma* or leukemia* or leukaemia* or metastat* or sarcoma* or teratoma*).tw,kf.
4	2 or 3
5	survivors/
6	survivor*.tw,kf.
7	5 or 6
8	(4 and 7) or 1
9	Food Habits/ or Food Preferences/ or exp Food/ or Eating/
10	Diet/ or Diet, Healthy/ or exp Nutrition Therapy/ or exp dietary proteins/ or exp dietary supplements/
11	(diet* or nutrition* or nutrient* or food* or feed* or eat* or drink*).tw,kf.
12	(fat* or carbohydrate* or (Diet* adj2 protein*) or fruit* or vegetable* or fibre* or fiber* or fish* or meat* or poultry or dairy or salt* or sugar* or cereal* or nut* or seed* or alcohol* or caffeine).tw,kf.
13	(macrobiotic or ketogenic or vegetarian or (low adj (glycemic* or glycaemic*))).tw,kf.
14	((food or diet or eating*) adj3 (behaviour* or practice* or choice or preference* or intake or consumption)).tw,kf.
15	(appetite* or taste* or flavor).tw,kf.
16	exercis*.tw,kf.
17	exp Exercise Therapy/ or exp Physical Therapy Modalities/ or Rehabilitation/
18	exp Exercise/ or sedentary behavior/ or healthy lifestyle/
19	movement/ or exp muscle strength/ or exp physical endurance/ or physical exertion/ or exp physical fitness/ or exp postural balance/ or exp posture/
20	exp Sports/ or Yoga/
21	Dance Therapy/
22	((resistance or strength or weight) adj2 train*).tw,kf.
23	(physical fitness or sport* or physical activit* or walk* or jog* or swim* or bicycl* or bike or danc* or aerobic exercise* or yoga or physiotherap* or rehab*).tw,kf.
24	((exercise or "physical activit*") adj3 (behaviour* or practice* or choice or preference* or intake or consumption)).tw,kf.
25	or/9-24
26	Attitude to Health/
27	Health Knowledge, Attitudes, Practice/ or Health Behavior/ or Health Education/
28	Patient Education as Topic/ or Health Literacy/
29	exp Motivation/
30	Perception/

31	("knowledge attitude* or practice*" or literac* or motivat* or educat* or opinion* or perception* or behavior*).tw,kf.
32	or/26-31
33	8 and 25 and 32
34	limit 33 to yr="2003 -Current"

DATABASE: Emcare

#	Searches
1	cancer survivor/
2	exp neoplasm/
3	(cancer* or tumor* or tumour* or neoplas* or malignan* or carcinoma* or adenocarcinoma* or choriocarcinoma* or leukemia* or leukaemia* or metastat* or sarcoma* or teratoma*).tw,kf.
4	2 or 3
5	survivor/
6	survivor*.tw,kf.
7	5 or 6
8	(4 and 7) or 1
9	feeding behavior/ or eating habit/ or food preference/ or exp food/ or exp food intake/
10	healthy diet/ or diet/ or diet therapy/ or dietary supplement/ or protein intake/ or Nutrition/
11	(diet* or nutrition* or nutrient* or food* or feed* or eat* or drink*).tw,kf.
12	(fat* or carbohydrate* or (Diet* adj2 protein*) or fruit* or vegetable* or fibre* or fiber* or fish* or meat* or poultry or dairy or salt* or sugar* or cereal* or nut* or seed* or alcohol* or caffeine).tw,kf.
13	(macrobiotic or ketogenic or vegetarian or (low adj (glycemic* or glycaemic*))).tw,kf.
14	((food or diet or eating*) adj3 (behaviour* or practice* or choice or preference* or intake or consumption)).tw,kf.
15	(appetite* or taste* or flavor).tw,kf.
16	exercis*.tw,kf.
17	exp kinesiotherapy/ or exp physiotherapy/ or rehabilitation/
18	exp exercise/ or sedentary lifestyle/ or healthy lifestyle/ or "movement (physiology)"/ or exp muscle strength/ or endurance training/ or fitness/ or exp body equilibrium/ or exp body position/ or exp sport/ or yoga/ or Dance Therapy/
19	((resistance or strength or weight) adj2 train*).tw,kf.
20	(physical fitness or sport* or physical activit* or walk* or jog* or swim* or bicycl* or bike or danc* or aerobic exercise* or yoga or physiotherap* or rehab*).tw,kf.
21	((exercise or "physical activit*") adj3 (behaviour* or practice* or choice or preference* or intake or consumption)).tw,kf.
22	or/9-21
23	attitude to health/ or attitude/ or health behavior/ or knowledge/
24	health education/ or health literacy/ or nutrition education/ or patient education/
25	patient education/
26	exp motivation/

27	perception/
28	("knowledge attitude* or practice*" or literac* or motivat* or educat* or opinion* or perception* or behavior*).tw,kf.
29	or/23-28
30	8 and 22 and 29
31	limit 30 to yr="2003 -Current"

DATABASE: CINAHL

Search Terms
Limiters - Published Date: 20030101-20231231<br><br>Expanders - Apply equivalent subjects<br><br>Search modes - Boolean/Phrase
S9 AND S25 AND S29
S26 OR S27 OR S28
TI (("knowledge attitude* or practice*" or literac* or motivat* or educat* or opinion* or perception* or behavior*)) OR AB (("knowledge attitude* or practice*" or literac* or motivat* or educat* or opinion* or perception* or behavior*))
(MH "Health Knowledge") OR (MH "Health Behavior") OR (MH "Patient Education") OR (MH "Health Literacy") OR (MH "Motivation+") OR MH "Perception")
(MH "Attitude to Health")
S10 OR S11 OR S12 OR S13 OR S14 OR S15 OR S16 OR S17 OR S18 OR S19 OR S20 OR S21 OR S22 OR S23 OR S24
TI (((exercise or "physical activit*") N3 (behaviour* or practice* or choice or preference* or intake or consumption))) OR AB (((exercise or "physical activit*") N3 (behaviour* or practice* or choice or preference* or intake or consumption)))
TI ((physical fitness or sport* or physical activit* or walk* or jog* or swim* or bicycl* or bike or danc* or aerobic exercise* or yoga or physiotherap* or rehab*)) OR AB ((physical fitness or sport* or physical activit* or walk* or jog* or swim* or bicycl* or bike or danc* or aerobic exercise* or yoga or physiotherap* or rehab*))
TI (((resistance or strength or weight) N2 train*)) OR AB (((resistance or strength or weight) N2 train*))
(MH "Dance Therapy")
(MH "Physical Fitness+") OR (MH "Balance, Postural") OR OR (MH "Posture") OR (MH "Yoga Pose") OR (MH "Balance Training, Physical") OR (MH "Yoga") OR (MH "Life Style, Sedentary")
(MH "Exercise+") OR (MH "Muscle Strengthening+") OR (MH "Upper Extremity Exercises+") OR (MH "Walking+") OR (MH "Physical Fitness") OR (MH "Sports+") OR (MH "Aerobic Exercises") OR (MH "Physical Endurance+") OR (MH "Exercise Physiology")
(MH "Therapeutic Exercise+") OR (MH "Physical Therapy+") OR (MH "Rehabilitation")
TI exercis* OR AB exercis*
TI (((food or diet or eating*) N3 (behaviour* or practice* or choice or preference* or intake or consumption))) OR AB (((food or diet or eating*) N3 (behaviour* or practice* or choice or preference* or intake or consumption)))
TI ((appetite* or taste* or flavor)) OR AB ((appetite* or taste* or flavor))

TI ((macrobiotic or ketogenic or vegetarian or (low N1 (glycemic* or glycaemic*)))) OR AB ((macrobiotic or ketogenic or vegetarian or (low N1 (glycemic* or glycaemic*))))
TI (fat* or carbohydrate* or (Diet* N2 protein*) or fruit* or vegetable* or fibre* or fiber* or fish* or meat* or poultry or dairy or salt* or sugar* or cereal* or nut* or seed* or alcohol* or caffeine) OR AB (fat* or carbohydrate* or (Diet* N2 protein*) or fruit* or vegetable* or fibre* or fiber* or fish* or meat* or poultry or dairy or salt* or sugar* or cereal* or nut* or seed* or alcohol* or caffeine)
TI (diet* or nutrition* or nutrient* or food* or feed* or eat* or drink*) OR AB (diet* or nutrition* or nutrient* or food* or feed* or eat* or drink*)
(MH "Diet+") OR (MH "Food Intake") OR (MH "Diet Therapy+") OR (MH "Dietary Proteins+") OR (MH "Dietary Supplements+")
(MH "Food Habits") OR (MH "Health Food") OR (MH "Food+") OR (MH "Functional Food") OR (MH "Access to Healthy Foods") OR (MH "Food Preferences")
S1 OR S8
S4 AND S7
S5 OR S6
TI survivor* OR AB survivor*
(MH "Survivors")
S2 OR S3
TI ((cancer* or tumor* or tumour* or neoplas* or malignan* or carcinoma* or adenocarcinoma* or choriocarcinoma* or leukemia* or leukaemia* or metastat* or sarcoma* or teratoma*)) OR AB ((cancer* or tumor* or tumour* or neoplas* or malignan* or carcinoma* or adenocarcinoma* or choriocarcinoma* or leukemia* or leukaemia* or metastat* or sarcoma* or teratoma*))
(MH "Neoplasms+")
(MH "Cancer Survivors")

DATABASE: Scopus

(TITLE-ABS ("knowledge attitude* or practice*" OR literac* OR motivat* OR educat* OR opinion* OR perception* OR behavior*)) AND (TITLE-ABS (diet* OR nutrition* OR nutrient* OR food* OR feed* OR eat* OR drink* OR fat* OR carbohydrate* OR (diet* W/2 protein*) OR fruit* OR vegetable* OR fibre* OR fiber* OR fish* OR meat* OR poultry OR dairy OR salt* OR sugar* OR cereal* OR nut* OR seed* OR alcohol* OR caffeine OR macrobiotic OR ketogenic OR vegetarian OR (low W/1 (glycemic* OR glycaemic*)) OR ((food OR diet OR eating*) W/3 (behaviour* OR practice* OR choice OR preference* OR intake OR consumption)) OR appetite* OR taste* OR flavor OR exercis* OR ((resistance OR strength OR weight) W/2 train*) OR "physical fitness" OR sport* OR "physical activit*" OR walk* OR jog* OR swim* OR bicycl* OR bike OR danc* OR aerobic AND exercise* OR yoga OR physiotherap* OR rehab* OR ((exercise OR "physical activit*") W/3 (behaviour* OR practice* OR choice OR preference* OR intake OR consumption)))) AND (TITLE-ABS ("cancer survivor*" OR (survivor* AND (cancer* OR tumor* OR tumour* OR neoplas* OR malignan* OR carcinoma* OR adenocarcinoma* OR choriocarcinoma* OR leukemia* OR leukaemia* OR metastat* OR sarcoma* OR teratoma*))))
